# Total Synthesis of (+)‐Disorazole Z1

**DOI:** 10.1002/chem.202501452

**Published:** 2025-05-29

**Authors:** Thomas J. Bauer, Sooroosh Aminian, Oliver Spieß, Dieter Schinzer

**Affiliations:** ^1^ Chemisches Institut Otto‐von‐Guericke‐Universität Universitätsplatz 2 Magdeburg 39106 Germany

**Keywords:** anti‐cancer agent, consecutive aldol reactions, cytotoxicity, lactone opening, total synthesis

## Abstract

We wish to report the first total synthesis of (+)‐disorazole Z1, a biologically extremely potent natural product (picomolar cytotoxicity) with complex molecular architecture featuring a 26‐membered macrodiolide with lateral chains including a quaternary chiral carbon center. The complex acyclic lateral chains containing a stereochemical triad with the quaternary center surrounded by two hindered secondary alcohols were synthesized in a straightforward way by the use of two consecutive and highly diastereoselective aldol reactions with a lactone intermediate and further transformations to yield the desired (*Z*)‐vinyl iodide. Successful esterification with a functionalized diene‐stannane yielded the fully elaborated carbon skeleton. Making use of the C2 symmetry of the natural product, a double Stille coupling gave the protected natural product. Final deprotection provided (+)‐disorazole Z1, which was fully identical to a natural sample.

## Graphical abstract

The first total synthesis of disorazole Z1, a highly up to picomolar active cytotoxic natural product, is presented. An acyclic carbon chain containing a chiral quaternary carbon center surrounded by two chiral secondary alcohols was synthesized and combined with a functionalized diene‐stannane to obtain the carbon skeleton of one half of disorazole Z1. Fitting two identical halves together resulted in the synthetic natural product.

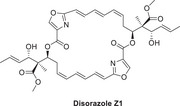

## 1 Introduction

The disorazoles are a family of 39 macrodiolides isolated so far, showing macrolide ring sizes between 26 and 32.^[^ ^1^, ^2^ ^]^ In a very recent paper by R. Müller et al., a whole family of disorazoles of the Z‐type (Z1 – Z10) has been described, and their biology has been evaluated.^[^ ^3^ ^]^ Based on fermentation data, disorazole Z1 is the major component of the disorazole Z family.^[^ ^4^ ^]^ All disorazoles are secondary metabolites from myxobacterium cellulosum. 29 variants of them are produced by strain So ce 12 and have been isolated by the research groups of Höfle and Reichenbach in 1994.^[^ ^5^ ^]^ Members of the disorazole Z family were found in the culture broths of strains So ce 1875 and 427. Very recently, a comprehensive updated account on the chemistry and biology of disorazoles was published by K.‐H. Altmann et al.^[^ ^6^ ^]^

**Figure 1:**
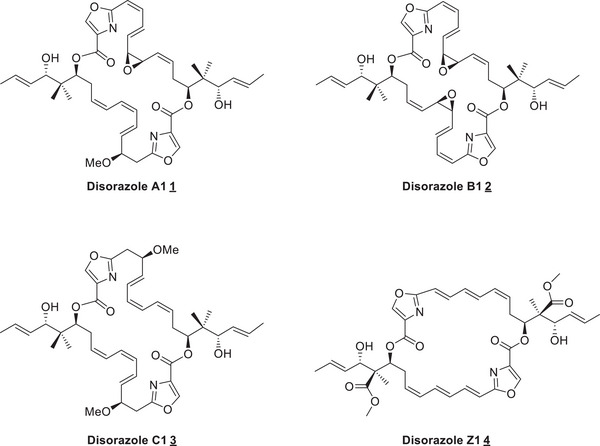
Selected members of the disorazole family.

All disorazoles show very potent antitumor activity due to inhibition of tubulin polymerization combined with a very powerful cytotoxicity up to picomolar activity against various human cancer cell lines. This exciting biological profile generated a tremendous interest in the scientific community in both total synthesis and biology. In addition, the enormous biological potency makes them very attractive in personalized medicine as potential payloads for antibody‐drug conjugates (ADCs) in targeted cancer therapy (Figure 1).^[^ ^7^ ^]^

Along these lines we already published a flexible and robust new route to synthesize (‐)‐disorazole C1 **3**, which involved in the final steps a coupling of the building blocks via Yamaguchi esterification and a final Yamaguchi macrolactonization to obtain the macrocycle.^[^ ^8^ ^]^ Furthermore, a series of interesting disorazole analogues were published in order to help the understanding of the binding mode to tubulin of these fascinating natural products providing great chances to study structure‐activity relationships (SAR).^[^ ^9^, ^10^ ^]^

In this communication, we wish to disclose the first total synthesis of (+)‐disorazole Z1, an extremely biologically active member of the disorazole family showing up to picomolar cytotoxic activity through targeting cancer cells by inhibiting tubulin polymerization and destabilizing microtubules. In addition, disorazole Z1 activates dendritic cells and increases the immune effector T cell activity by affecting the dynamic phosphorylation of ezrin, a key mediator of immune receptor activity.^[^ ^11^, ^12^, ^13^ ^]^

**Scheme 1:**
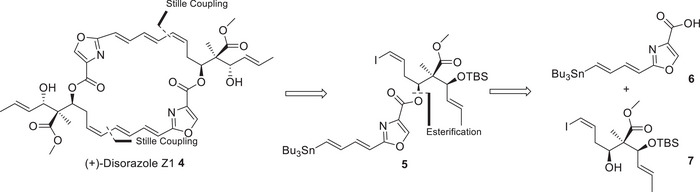
Retrosynthetic analyzes of disorazole Z1.

As depicted in Scheme 1 our general retrosynthetic analysis follows Altmann´s approach but with the demand to establish the quaternary carbon center in intermediate **7**.^[^ ^14^ ^]^

Besides the highly promising biological profile of disorazole Z1, it is a very challenging target for total synthesis because it contains a chiral quaternary carbon center at C13, which is only present in the disorazole Z family. Several research groups already focused their efforts on a synthetic approach to tackle this complex molecular architecture. However, to the best of our knowledge, no total synthesis of any disorazole Z member has been disclosed until now. M. Kalesse et al.^[^ ^15^ ^]^ reported the first synthesis of the disorazole Z core with reduced complexity by the use of a gem‐dimethyl unit instead of the chiral quaternary carbon center, and very recently K.‐H. Altmann et al. communicated the synthesis of a bis(desmethyl) derivative of disorazole Z1 with the methylester unit present but missing the methyl group at C13. In general, all these synthesized analogues displayed a striking biological activity, and they are important molecules to study SAR.

## 2 Results and Discussion

**Scheme 2:**
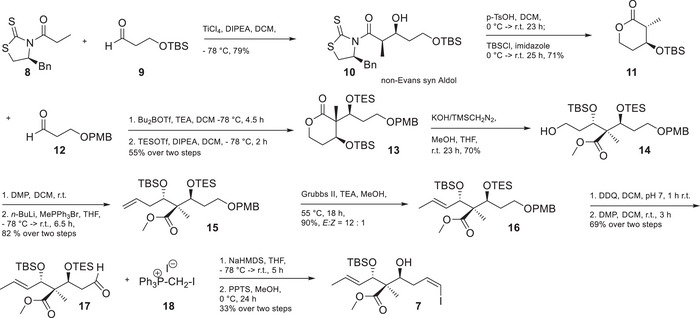
Synthesis of vinyl iodide **7**. (DDQ, 2,3‐dichloro‐5,6‐dicyano‐1,4‐benzoquinone; DMP, Dess‐Martin periodinane; PPTS, pyridinium *p*‐toluenesulfonate).

The overall strategy of our synthesis to construct the chiral quaternary center makes use of two highly diastereoselective aldol reactions followed by lactone opening yielding the desired stereochemical triad in a very efficient way (Scheme 2).

Starting with the known (*S*)‐configured thiazolidinethione **8,** a diastereoselective aldol reaction with aldehyde **9** in the presence of titanium tetrachloride was performed at ‐78 °C to yield the desired non‐Evans *syn* aldol product **10** in 79% yield.^[^ ^16^, ^17^ ^]^ In the presence of *p*‐toluenesulfonic acid monohydrate and subsequent protection of the alcohol function, the chiral lactone **11** was obtained in 71% yield. With lactone **11** in hand, we could perform the second aldol reaction this time with a PMB‐protected propanal **12** reacting with a pre‐formed boron enolate of **11**.^[^ ^18^ ^]^ Again, the required aldol product **13** was obtained after immediate protection in 55% yield over two steps. Thereby, the final correct (*S*) configuration of the quaternary center still masked in the lactone moiety was established. Finally, a mild lactone opening in the presence of KOH in methanol and in situ esterification with TMSCH_2_N_2_ solution led to the fully functionalized acyclic stereochemical triad **14** with all four alcohols nicely differentiated for the necessary final operations.

Simple DMP oxidation followed by a Wittig reaction yielded the one‐carbon extended terminal olefin **15** in 82% yield over two steps. Next, a very efficient isomerization catalyzed by the Grubbs II catalyst, based on the method of Hanessian et al., provided the required (*E*)‐olefin **16** in 90% yield with a 12 : 1 = *E* : *Z* ratio.^[^ ^19^ ^]^ DDQ‐promoted oxidative cleavage^[^ ^20^ ^]^ of the PMB‐protected alcohol, followed again by DMP oxidation gave aldehyde **17** in 69% yield over both steps. A Stork‐Zhao‐Wittig olefination with (iodomethyl)triphenylphosphonium iodide **18** and NaHMDS as the base employed in THF offered the needed (*Z*)‐iodide, which was directly deprotected with PPTS as the crude olefination product to yield alcohol **7** in pure form in 33% over two steps. Compound **7** represents the fully functionalized acyclic fragment consisting of the naturally configured stereochemical triad with the quaternary carbon center.^[^ ^21^ ^]^

**Scheme 3:**
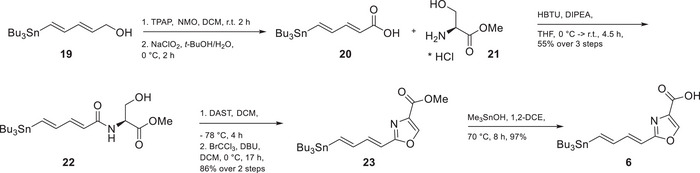
Synthesis of stannane **6**. (DAST, diethylaminosulfur trifluoride; DBU, 1,8‐diazabicyclo[5.4.0]undec‐7‐ene; HBTU, hexafluorophosphate benzotriazole tetramethyl uronium).

Next, the required (*E*,*E*)‐diene‐stannane **6** containing the required oxazole‐acid was also synthesized in a straightforward way, shortening Altmann´s protocol (scheme 3). It was significantly streamlined to reduce the total number of synthetic steps and simplify the overall process, and all in all, ultimately saving time to prepare the second key fragment of the synthesis. In the original protocol used by Altmann et al.,^[^ ^14^ ^]^ the synthesis was initiated from ethynyltrimethylsilane and proceeded through a six‐step sequence to afford dienoic acid **20**, which was subsequently coupled to (trimethylsilyl)ethyl protected serine, an intermediate that was prepared through multiple steps involving protection and deprotection strategies. To streamline the process, the synthesis of the protected amino alcohol was bypassed, and instead commercially available *L*‐serine methyl ester hydrochloride **21** was used as the amine coupling partner. This modification allowed for direct amide bond formation with the stannyl‐dienoic acid **20**, prepared from alcohol **19**, under standard peptide coupling conditions, affording the corresponding amide **22**.

Subsequent cyclodehydration of the secondary amide was carried out to furnish the corresponding dihydrooxazole intermediate, which was then oxidized to the oxazole core **23**. Finally, the methyl ester was hydrolyzed using trimethyltin hydroxide following the procedure reported by K.C. Nicolaou et al.^[^ ^22^ ^]^ Overall, the modified synthetic approach was shown to enhance operational simplicity, eliminate the need for protecting group manipulations, and improve overall time efficiency while delivering the desired product in high quality.

**Scheme 4:**
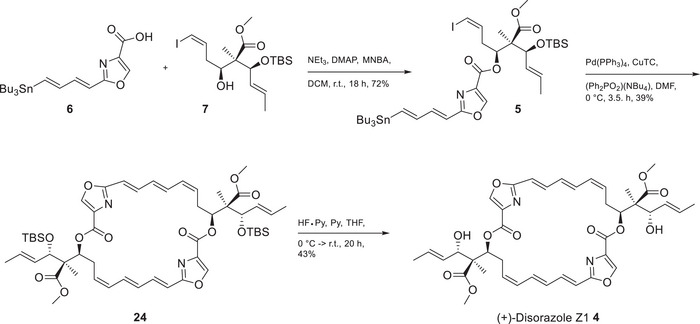
Final synthesis of (+)‐disorazole Z1 **4**. (CuTC, copper(I) thiophene‐2‐carboxylate; MNBA, 2‐methyl‐6‐nitrobenzoic anhydride).

In a final sequence, alcohol **7** was esterified with acid **6** under exclusion of light and under argon in the presence of DMAP and Shiina reagent (MNBA) to provide the required ester **5** in 72% yield. Compound **5** represents the complete carbon skeleton of one half of the 26‐membered macrocycle, including the lateral chain with the three correctly configured chiral centers (Scheme 4).

The end game of the synthesis is based on Altmann´s successful strategy to envision a double Stille coupling (an intermolecular Stille coupling followed in situ by an intramolecular Stille coupling) as a powerful alternative to the Yamaguchi protocol to construct C2‐symmetric macrocycles. Based on Kalesse´s findings and our own experience in connection with the total synthesis of disorazole C1, the Yamaguchi strategy suffers from the instability of the cyclization precursors and requires a late hydrogenation approach to increase the stability for the ring closure.

Due to the C2‐symmetry of disorazole Z1 compound, **5** was cyclized under double‐Stille coupling conditions under Pd‐catalysis and CuTC as the co‐catalyst in dry DMF to obtain 39% of the TBS‐protected disorazole Z1 **24**. The final deprotection of the TBS‐ethers under standard conditions with HF pyridine in THF/pyridine and under light exclusion gave (+)‐disorazole Z1 **4** in 43% yield, which was identical in all analytical data with an authentic natural sample.^[^ ^23^ ^]^

## 3 Conclusion

In conclusion, we have presented the first total synthesis of (+)‐disorazole Z1 with a longest linear sequence of 15 steps. The major challenge of the synthesis was the stereoselective construction of the quaternary carbon center by the use of two highly diastereoselective consecutive aldol reactions with a lactone intermediate. This provided the fully functionalized acyclic fragment with three contiguous chiral centers. The second crucial intermediate of the synthesis furnished the diene‐stannane with the desired oxazole core, which was obtained in a shortened route. Esterification of the two fragments delivered the complete carbon skeleton of disorazole Z1, which was cyclized under double Stille cyclization conditions to reach the TBS‐protected macrocyclic core of disorazole Z1. Removal of the TBS protecting groups gave (+)‐disorazole Z1, which was identical in all aspects with an authentic sample.

In summary, our flexible and robust strategy to construct **4** offers also great opportunities to generate a large variety of analogues to study SAR to gain more insights into this fascinating disorazole Z family of natural products displaying extremely potent biological activity.

## Supporting Information

The authors have cited additional references within the Supporting Information.^[^ ^24^, ^25^, ^26^, ^27^, ^28^, ^29^, ^30^, ^31^ ^]^ The supporting information includes experimental procedures and analytical data.

## Conflict of Interests

The authors declare no conflict of interest.

## Supporting information



Supporting information

## Data Availability

The data that support the findings of this study are available in the supplementary material of this article.

## References

[1] Jahresbericht GBF 2001. Available online: https://www.helmholtz‐hzi.de/fileadmin/user_upload/Infothek/Ueber_das_HZI/Jahresberichte/Ergebnisberichte/Annual_Report_2001.pdf (accessed on 7 February 2024).

[2] H. Irschik , R. Jansen , K. Gerth , G. Höfle , H. Reichenbach , J. Antibiot. 1995, 48, 31. 10.7164/antibiotics.48.31 7868386

[3] Y. Gao , J. Birkelbach , C. Fu , J. Herrmann , H. Irschik , B. Morgenstern , K. Hirschfelder , R. Li , Y. Zhang , R. Jansen , R. Müller , Microbiol. Spectr. 2023, 11, e00730‐23. 37318329 10.1128/spectrum.00730-23 PMC10434194

[4] H. Irschik , R. Jansen , F. Sasse , EP 1743897A1, 2005.

[5] R. Jansen , H. Irschik , V. Reichenbach , G. Wray , G. Höfle , Liebigs Ann. 1994, 759.

[6] For a review of the literature up to 2008, see: C. D. Hopkins , P. Wipf , Nat. Prod. Rep. 2009, 26, 585, very recent update: C. P. Bold, K.‐H. Altmann *Tetrahedron* **2024**, 133908.19387496

[7] H. L. Perez , P. M. Cardaerelli , S. Deshpande , S. Gangwar , G. M. Schroeder , G. D. Vite , R. M. Borzilleri , Drug Discovery Today 2014, 19, 869. 24239727 10.1016/j.drudis.2013.11.004

[8] L. Lizzadro , O. Spieß , D. Schinzer , Org. Lett. 2021, 23, 4543. 34037403 10.1021/acs.orglett.1c01123

[9] L. Lizzadro , O. Spieß , W. Collisi , L. Stadler , D. Schinzer , ChemBioChem 2022, 23, e202200458. 35998215 10.1002/cbic.202200458 PMC9826379

[10] L. Lizzadro , O. Spieß , S. Reinecke , M. Stadler , D. Schinzer , Molecules 2024, 29 1123. 38474635 10.3390/molecules29051123 PMC10934378

[11] K. Rox , M. Rohde , G. S. Chhatwal , R. Müller , Cell Chem. Biol. 2017, 24, 159. 28089757 10.1016/j.chembiol.2016.12.011

[12] K. Rox , M. Rohde M , G. S. Chhatwal , R. Müller , D. Pore , N. Gupta , Crit. Rev. Immunol. 2015, 35 15. 25746045 10.1615/critrevimmunol.2015012327 PMC4548853

[13] X. Wan , A. Mendoza , C. Khanna , L. J. Helman , Cancer Res. 2005, 65 2406. 15781656 10.1158/0008-5472.CAN-04-3135

[14] C. P. Bold , D. Lucena‐Agell , M. Á Oliva , J. F. Diaz , K.‐H. Altmann , Angew. Chem. Int. Ed. 2023, 62, e202212190. 10.1002/anie.202212190 PMC10107878 36281761

[15] R. Schäckel , B. Hinkelmann , F. Sasse , M. Kalesse , Angew. Chem. Int. Ed. 2010, 49, 1619. 10.1002/anie.200906450 20099286

[16] M. T. Crimmins , B. W. King , E. A. Tabet , K. Chaudhary , J. Org. Chem. 2001, 66, 894. 11430110 10.1021/jo001387r

[17] J. Wang , R. P. Hsung , S. K. Ghosh , Org. Lett. 2004, 6, 1939. 15176788 10.1021/ol0495624

[18] F. Weber , F. Becker , M. Keller , H. Hillebrecht , R. Brückner , Eu. J. Org. Chem. 2015, 7892.

[19] S. Hanessian , S. Giroux , A. Larsson , Org. Lett. 2006, 8, 5481. 17107052 10.1021/ol062167o

[20] I. Paterson , O. Delgado , G. J. Florence , I. Lyothier , M. O'Brien , J. P. Scott , N. Sereinig , J. Org. Chem. 2005, 70, 150. 15624917 10.1021/jo048534w

[21] T. J. Bauer planned PhD thesis, OVGU Magdeburg, 2025.

[22] K. C. Nicolaou , A. A. Estrada , M. Zak , S. H. Lee , B. S. Safina , Angew. Chem. Int. Ed. 2005, 44, 1378. 10.1002/anie.200462207 15674985

[23] S. Aminian planned PhD thesis, OVGU Magdeburg, 2025.

[24] S. P. Chavan , K. R. Harale , Tetrahedron Lett. 2012, 53, 4683.

[25] C. Herb , M. E. Maier , J. Org. Chem. 2003, 68, 8129. 14535794 10.1021/jo035054g

[26] S. J. Hein , D. Lehnherr , W. R. Dichtel , Chem. Sci. 2017, 8, 5675. 28989606 10.1039/c7sc01625e PMC5621055

[27] C. Amatore , E. Blart , J. P. Genet , A. Jutand , S. Lemaire‐Audoire , M. Savignac , J. Org. Chem. 1995, 60, 6829.

[28] P. S. Ng , R. W. Bates , Tetrahedron 2016, 72, 6356.

[29] H. Goossens , J. M. Winne , S. Wouters , L. Hermosilla , P. J. De Clercq , M. Waroquier , V. Van Speybroeck , S. Catak , J. Org. Chem. 2015, 80, 2609. 25615563 10.1021/jo5027639

[30] A. De Lera , V. Dominguez , US 6534545 B1, 2003.

[31] N. Huwyler , K. Radkowski , S. M. Rummelt , A. Fürstner , Chem. Eur. J. 2017, 23, 12412. 28714542 10.1002/chem.201702470

