# Supplementary material for: Total Synthesis of (+)‐Disorazole Z1
Source: Chemistry. 2025 May 29;31(38):e202501452. doi: 10.1002/chem.202501452 (PMC12238908; doi:10.1002/chem.202501452)
Supplement: Supplementary file 1 — Supporting information [file CHEM-31-e202501452-s001.docx]

**SUPPORTING INFORMATION**

**Table of Contents**

General……………………….………………………………......................................................S2

Experimental Procedures, ^1^H and ^13^C NMR spectra……..……………………………………….S3

**General**

Solvents were dried by standard procedures and redistilled under N_2_ atmosphere prior to use or were purchased in an appropriate water free quality and used as obtained. All reactions were run under nitrogen unless otherwise stated. For reactions that require heating, an oil bath was used. The products were purified by flash chromatography on Merck silica gel 60 (40-63 µm). POLYGRAM SIL G/UV254 prefabricated TLC plates with fluorescent indicator from Macherey-Nagel have been used for the analytical thin layer chromatography (TLC). The separated substances were detected by irradiation with UV light with a wavelength of 254 nm or staining with vanillin or potassium permanganate reagent and subsequent warming with a heat gun. Electrospray ionization (ESI) and electron ionisation (EI) mass spectra were recorded on Finnigan MAT 95 and Waters Xevo G2-TOF spectrometers. ^1^H and ^13^C NMR spectra were recorded on Brucker AVIII 400 and Brucker AVI 600 spectrometers. Chemical shifts (δ) are reported in ppm from tetramethylsilane, referenced to the solvent resonance resulting from incomplete deuteration (^1^H NMR = CDCl_3_: δ 7.26, CD_3_OD: δ 3.31; ^13^C NMR = CDCl_3_: δ 77.16, CD_3_OD: δ 49.00). Data are reported as follows: chemical shift, multiplicity (s = singlet, d = doublet, t = triplet, br = broad, m = multiplet, app = apparent), coupling constants (Hz) and integration. Optical rotations were recorded on an Anton Paar MCP150 polarimeter. Infrared (IR) spectra were recorded on Brucker Vertex 70v Bands are characterized as strong (s), medium (m), weak (w) or broad (br).

Scheme S1: Synthesis of the vinyl iodide **7**:


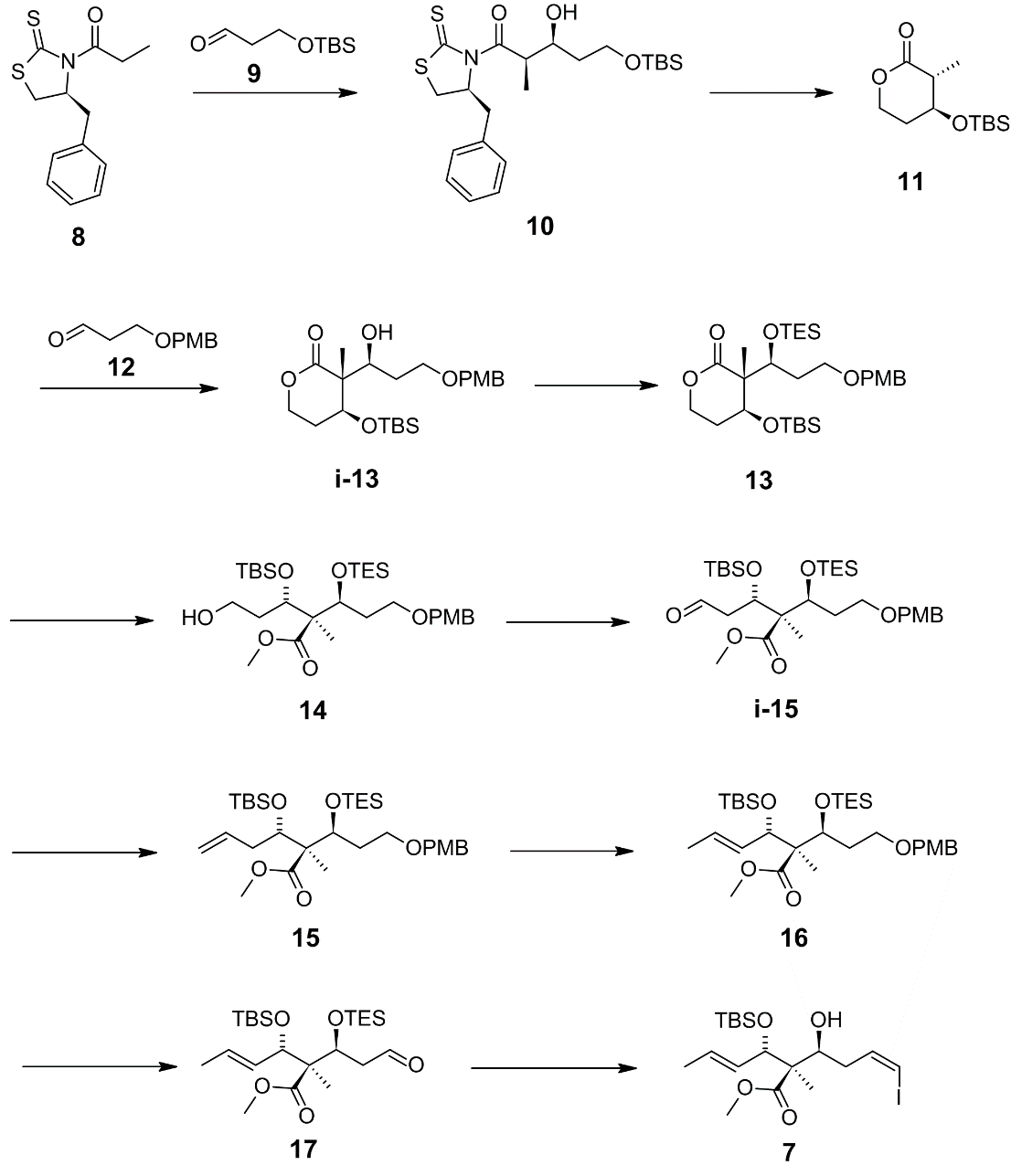


Scheme S2: Synthesis of the PMB-protected propanal **12**:


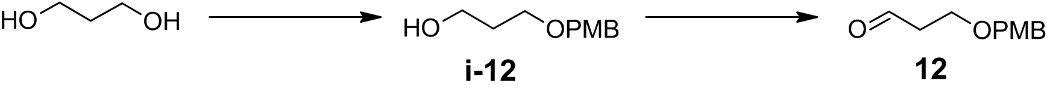


**(2*R*,3*S*)-1-((4*S*)-4-benzyl-2-thioxo-1,3-thiazolidin-3-yl)-**

**5-((*tert*-butyl(dimethyl)silyl)oxy)-3-hydroxy-2-methylpentan-1-one (10)**


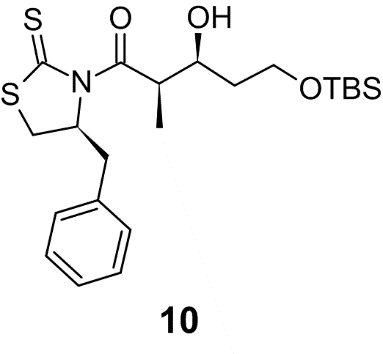


Under nitrogen atmosphere (*S*)-*N*-propionyl-thiazolidinethione (prepared see ref [16]) (**8**) (2 g, 7.55 mmol, 1.0 eq.) is dissolved in anhydrous dichloromethane (60 ml) and cooled to 0 °C. Titanium tetrachloride (0.96 ml, 8.74 mmol, 1.16 eq.) is added slowly and the orange suspension is stirred for 5 min. Then DIPEA (1.49 ml, 8.74 mmol, 1.16 eq.) is introduced slowly which leads to a red/black solution. After stirring for 20 min at 0 °C the reaction mixture is cooled to ‑78 °C. 3-((*tert*-butyl(dimethyl)silyl)oxy)propanal (prepared see ref [17]) (**9**) (1.7 g, 9.0 mmol, 1.2 eq.) is injected within two minutes and stirred for further 1 h at ‑78 °C. The reaction is quenched under vigorous stirring with saturated aqueous NH_4_Cl solution (28 ml) at ‑78 °C. While warming to room temperature water (28 ml) is poured in. The organic layer is separated and the aqueous layer is extracted with dichloromethane (3x50 ml). The combined organic layers are dried over MgSO_4_, filtered and the solvent is removed under reduced pressure. The residue is purified by column chromatography (n‑pentane/Et_2_O, 9:1) to afford product **10** (2.7 g, 5.95 mmol, 79 %, dr > 95:5) as a yellow sticky oil.

***R*_f_**=0.25 (n-pentane/Et_2_O 3:1); **[α]_D_^20^**=+143.985° (c=0.266 in CHCl_3_); **^1^H NMR** (600 MHz, CDCl_3_, ppm): δ=7.36-7.32 (m, 2H), 7.30-7.27 (m, 3H), 5.40 (dddd, *J*=10.5, 7.3, 4.0, 1.0 Hz, 1H), 4.70 (qd, *J*=7.0, 3.9 Hz, 1H), 4.28 (ddd, *J*=9.4, 3.9, 2.8 Hz, 1H), 3.87 (ddd, *J*=10.6, 6.0, 4.9 Hz, 1H), 3.82 (ddd, *J*=10.2, 7.7, 4.6 Hz, 1H), 3.43 (s, 1H), 3.36 (ddd, *J*=11.5, 7.2, 1.0 Hz, 1H), 3.24 (dd, *J*=13.2, 4.0 Hz, 1H), 3.04 (dd, *J*=13.2, 10.5 Hz, 1H), 2.88 (dd, *J*=11.5, 1.0 Hz, 1H), 1.80 (dddd, *J*=14.2, 9.4, 7.7, 4.9 Hz, 1H), 1.64 (dddd, *J*=14.1, 6.0, 4.6, 2.7 Hz, 1H), 1.24 (d, *J*=6.9, 3H), 0.90 (s, 9H), 0.08 (s, 6H); **^13^C NMR** (151 MHz, CDCl_3_, ppm): δ=201.5, 177.5, 136.6, 129.6, 129.0, 127.4, 71.0, 69.1, 62.0, 43.6, 37.2, 36.1, 31.9, 26.0, 18.4, 11.6, ‑5.30, ‑5.31; **IR** (neat, cm^-1^): ν^~^=3486 (w br), 3029 (w), 2951 (m), 2928 (m), 2881 (m), 2856 (m), 1686 (m), 1341 (m), 1252 (s), 1157 (s), 1136 (s), 1098 (s), 1080 (s), 1030 (m), 833 (s), 776 (s), 743 (s), 701 (s); **HRMS** (ESI): *m/z* calcd for C_22_H_36_NO_3_S_2_Si [*M*+H]^+^: 454.1906; found: 454.1899.


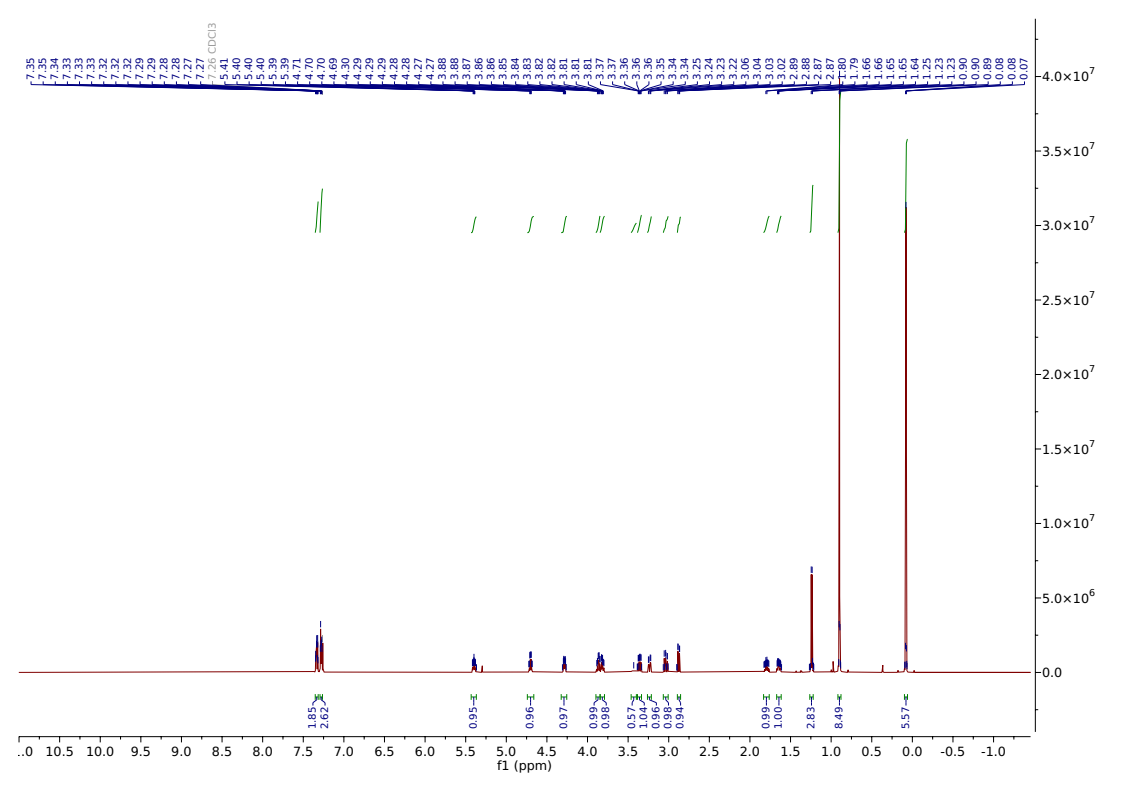

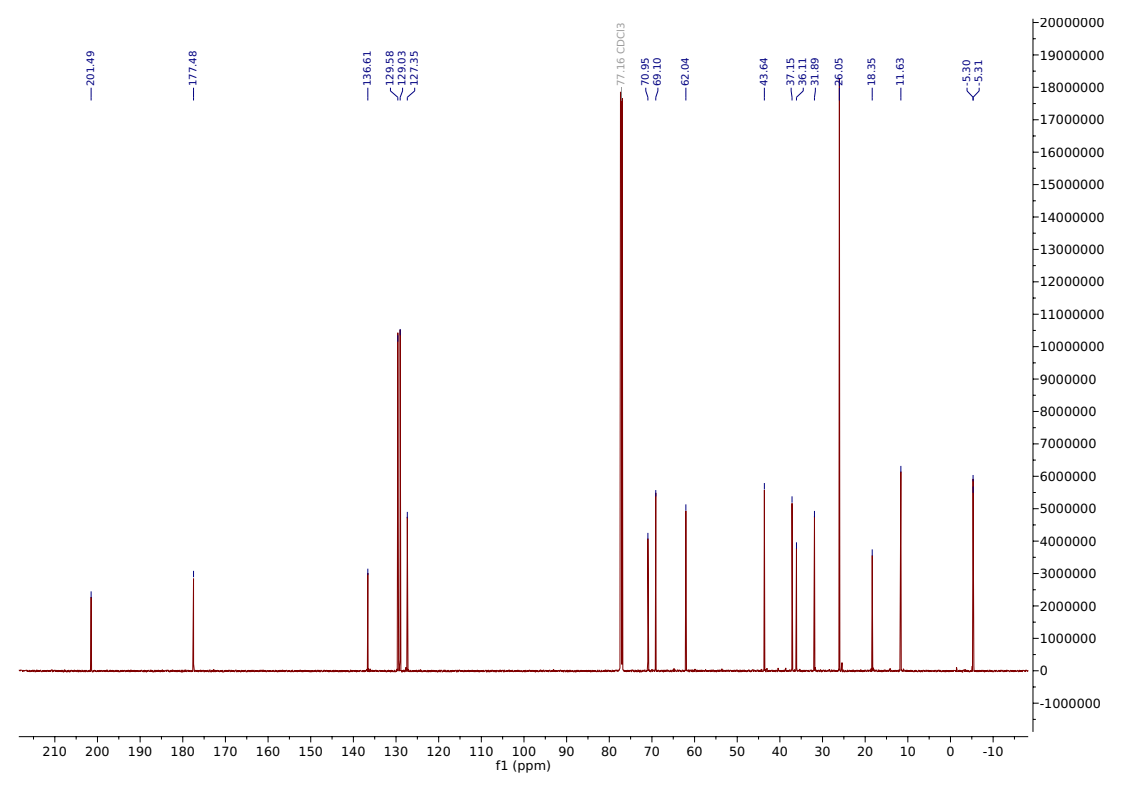

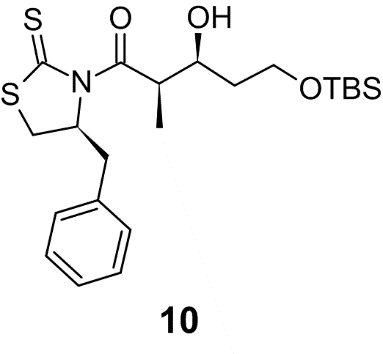


**(3*R*,4*S*)-4-((*tert*-butyl(dimethyl)silyl)oxy)-3-methyloxan-2-one (11)**


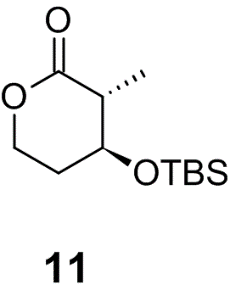


The aldol product **10** (2.595 g, 5.72 mmol, 1.0 eq.) is dissolved in dry dichloromethane (19.1 ml) and cooled to 0 °C. *Para*-toluenesulfonic acid monohydrate (598.4 mg, 3.146 mmol, 0.55 eq.) is added in portions and the solution is stirred for 1 h at 0 °C. After 23 h stirring at room temperature the colorless solution is again cooled to 0 °C and imidazole (1.17 g, 17.16 mmol, 3.0 eq.) and TBSCl (1.3 g, 8.58 mmol, 1.5 eq.) are introduced sequentially in portions. The reaction mixture is allowed to stir 10 min at 0 °C before it is warmed to room temperature and stirred for another 25 h. Under intense stirring saturated aqueous NaHCO_3_ solution (15 ml) and dichloromethane (20 ml) are added. The organic phase is separated and the aqueous layer is extracted with dichloromethane (3x20 ml). The combined organic layers are washed with water (16 ml), dried over MgSO_4_, filtered and concentrated under reduced pressure. After column chromatography (*n*‑pentane/Et_2_O, 6:1) the lactone **11** is provided (990 mg, 4.05 mmol, 71 %) as a colorless crystalline solid.

***R*_f_**=0.21 (n-pentane/Et_2_O 3:1); **m.p.**=75-76 °C; **[α]_D_^20^**=+27.636° (c=1.1 in CHCl_3_); **^1^H NMR** (400 MHz, CDCl_3_, ppm): δ=4.48 (ddd, *J*=11.3, 8.9, 4.0 Hz, 1H), 4.23 (ddd, *J*=11.3, 5.7, 4.7 Hz, 1H), 3.79 (td, *J*=6.3, 4.5 Hz, 1H), 2.54 (p, *J*=7.1 Hz, 1H), 2.12 (ddt, *J*=14.2, 9.0, 4.6 Hz, 1H), 1.80 (dtd, *J*=14.3, 5.8, 4.0 Hz, 1H), 1.29 (d, *J*=7.2 Hz, 3H), 0.89 (s, 9H), 0.08 (s, 3H), 0.07 (s, 3H); **^13^C NMR** (101 MHz, CDCl_3_, ppm): δ=174.1, 70.3, 64.9, 44.8, 31.4, 25.8, 18.0, 14.7, ‑4.4, ‑4.7; **IR** (neat, cm^-1^): ν^~^=2983 (w), 2953 (m), 2929 (m), 2886 (w), 2857 (m), 1738 (s), 1255 (s), 1114 (m), 1077 (s), 1040 (s), 1015 (m), 905 (s), 885 (s), 835 (s), 803 (s), 776 (s); **HRMS** (ESI): *m/z* calcd for C_12_H_25_O_3_Si [*M*+H]^+^: 245.1573; found: 245.1578.


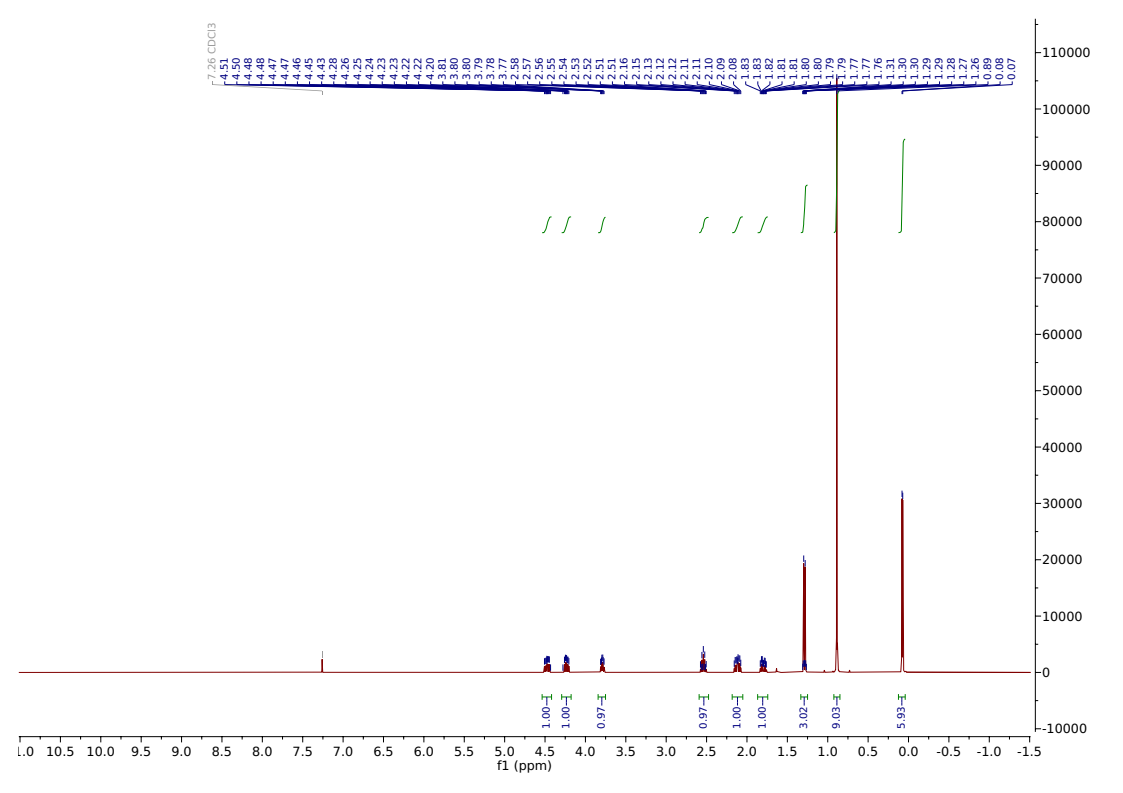

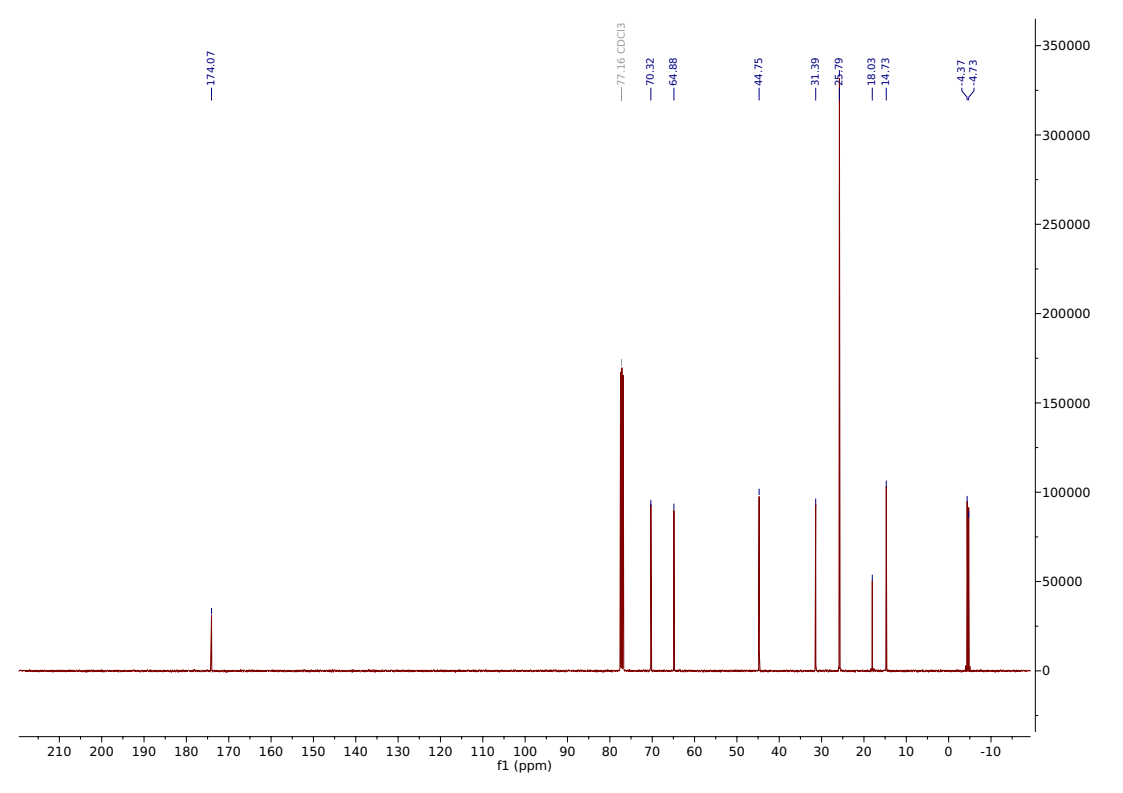

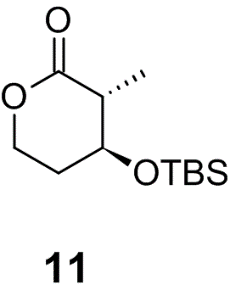


**(3*R*,4*S*)-4-((*tert*-butyl(dimethyl)silyl)oxy)-3-((1*S*)-1-hydroxy-**

**3-((4-methoxybenzyl)oxy)propyl)-3-methyloxan-2-one (i-13)**


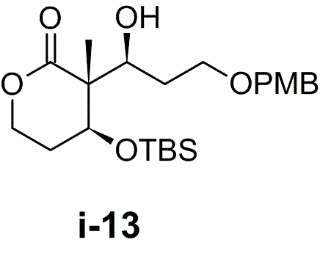


To a solution of lactone **11** (488 mg, 2.0 mmol, 1.0 eq.) in dry dichloromethane (9.2 ml) 1M Bu_2_BOTf in dichloromethane (2.2 ml, 2.2 mmol, 1.1 eq.) is injected at ‑78 °C under nitrogen atmosphere. After stirring of the yellow solution for 20 min at ‑78 °C triethylamine (0.36 ml, 2.6 mmol, 1.3 eq.) is added slowly which leads to a colorless solution. The reaction mixture is allowed to stir 1 h at this temperature. Then a mixture of 3‑((4‑methoxybenzyl)oxy)propanal (**12**) (777 mg, 4.0 mmol, 2.0 eq.) with anhydrous dichloromethane (1.0 ml) is added slowly dropwise within 20 min. After stirring of the colorless solution for 3 h at ‑78 °C the cold reaction mixture is quenched sequentially with 20 mM aqueous phosphate buffer (8 ml), methanol (4 ml) and 30 % H_2_O_2_ (2 ml) at ‑78 °C. The cooling bath is removed and vigorous stirring is continued for additional 1.5 h. Dichloromethane (10 ml) is added and the organic layer is separated. The aqueous layer is extracted with dichloromethane (3x20 ml) and the combined organic layers are washed once with an aqueous saturated Na_2_SO_3_ solution (14 ml). After drying over MgSO_4_, filtration and removing of the solvent the residue is purified by column chromatography (*n*‑pentane/Et_2_O, 2:1) to afford a mixture of the desired product **i-13** and an additional unknown isomer with a ratio of 4:1 (712 mg, 1.62 mmol, 81.2 %, dr = 4:1) as a colorless oil. An analytical sample was obtained by a further chromatographic purification.

***R*_f_**=0.43 (n-pentane/Et_2_O 1:3); **[α]_D_^20^**=+24.180° (c=0.488 in CHCl_3_); **^1^H NMR** (600 MHz, CDCl_3_, ppm): δ=7.24‑7.21 (m, 2H), 6.88‑6.85 (m, 2H), 4.48‑4.40 (m, 2H), 4.40‑4.37 (m, 1H), 4.33 (dd, *J*=8.8, 3.4 Hz, 1H), 4.23 (ddd, *J*=11.1, 8.5, 4.1 Hz, 1H), 3.88 (dd, *J*=10.5, 1.9 Hz, 1H), 3.80 (s, 3H), 3.72 (ddd, *J*=9.6, 5.5, 4.4 Hz, 1H), 3.60 (td, *J*=9.0, 3.8 Hz, 1H), 3.35 (s, 1H), 2.16 (dddd, *J*=14.7, 10.5, 8.8, 4.3 Hz, 1H), 2.03 (dddd, *J*=13.7, 6.1, 4.1, 3.4 Hz, 1H), 1.96‑1.87 (m, 1H), 1.79 (dddd, *J*=14.6, 5.7, 3.8, 1.9 Hz, 1H), 1.20 (s, 3H), 0.88 (s, 9H), 0.10 (s, 3H), 0.08 (s, 3H); **^13^C NMR** (151 MHz, CDCl_3_, ppm): δ=175.1, 159.4, 130.0, 129.5, 113.9, 75.0, 73.0, 69.6, 68.9, 65.2, 55.4, 54.1, 31.8, 29.5, 25.8, 18.1, 16.9, -4.2, ‑4.9; **IR**(neat, cm^-1^): ν^~^=3474 (w br), 2953 (m), 2930 (m), 2888 (w), 2856 (m), 1723 (s), 1612 (m), 1513 (s), 1463 (m), 1247 (s), 1172 (m), 1153 (m), 1085 (s), 1035 (s), 1006 (s), 936 (m), 834 (s), 775 (s); **HRMS** (ESI): *m/z* calcd for C_23_H_42_NO_6_Si [*M*+NH_4_]^+^: 456.2781; found: 456.2776.


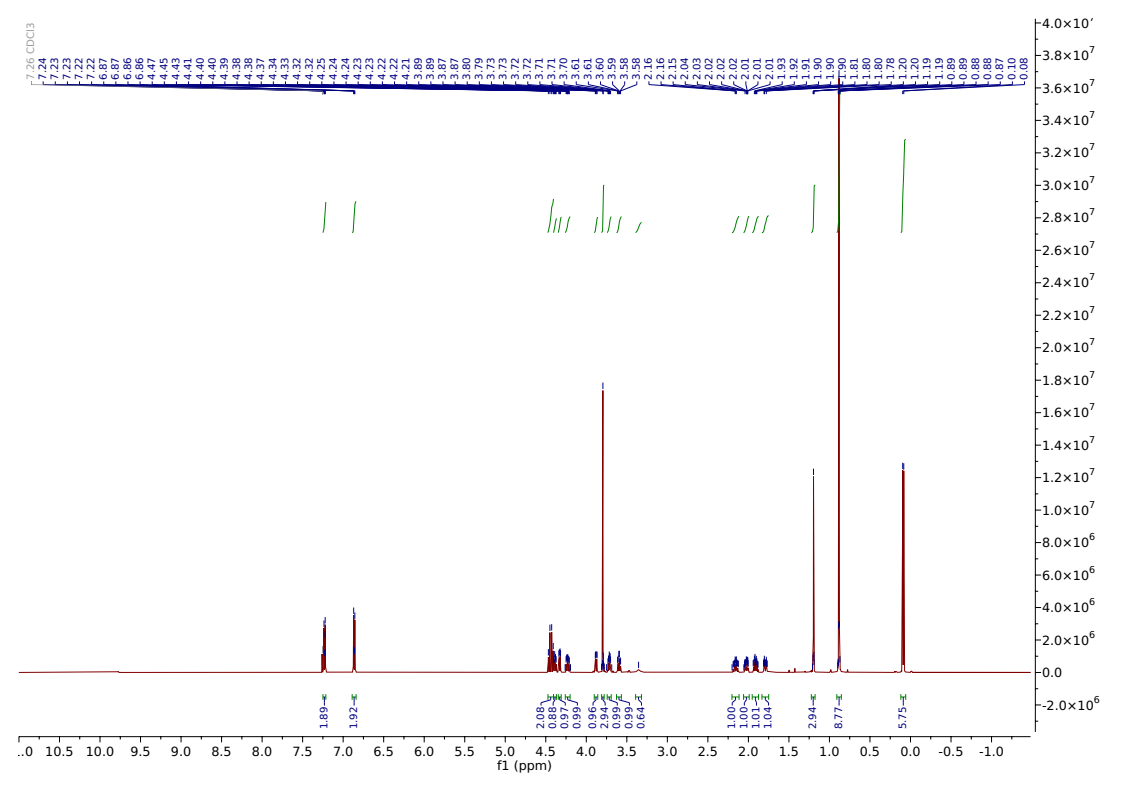

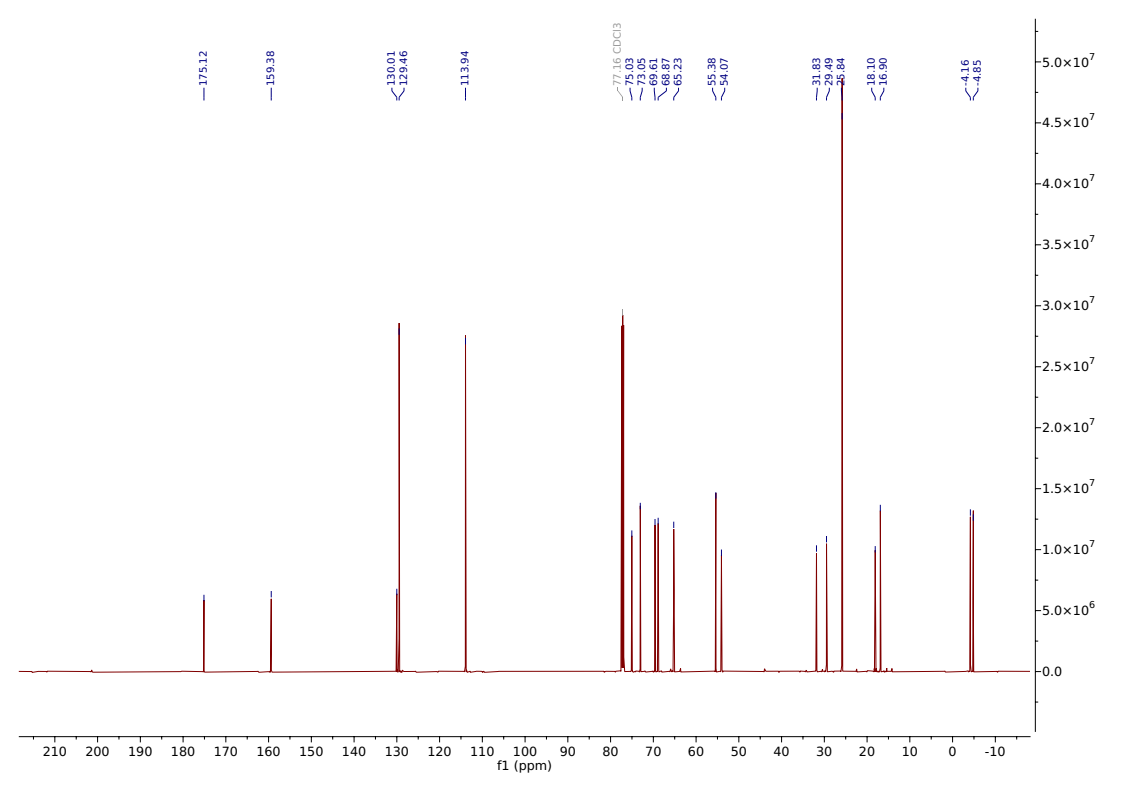

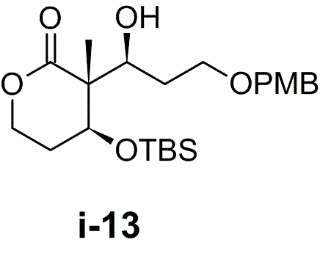


**(3*R*,4*S*)-4-((*tert*-butyl(dimethyl)silyl)oxy)-**

**3-((1*S*)-3-((4-methoxybenzyl)oxy)-1-((triethylsilyl)oxy)propyl)-**

**3-methyloxan-2-one (13)**


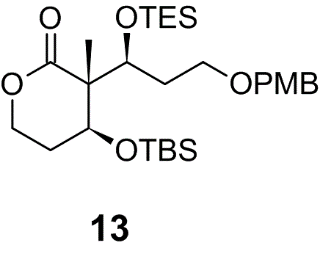


A solution of the previous isomeric mixture containing product **i-13** (630 mg, 1.44 mmol, 1.0 eq.) in anhydrous dichloromethane (5.3 ml) is cooled to -78 °C under nitrogen atmosphere. DIPEA (0.50 ml, 2.88 mmol, 2.0 eq.) and TESOTf (0.50 ml, 2.16 mmol, 1.5 eq.) are added sequentially. After stirring for 2 h at ‑78 °C the reaction mixture is quenched with a saturated aqueous NaHCO_3_ solution (3.5 ml) at ‑78 °C and diluted with dichloromethane (10 ml). The mixture is allowed to warm to ambient temperature, the organic phase is separated and the aqueous layer is extracted with dichloromethane (3x20 ml). The combined organic layers are dried over MgSO_4_, filtered and concentrated under reduced pressure. After column chromatography (*n*‑pentane/Et_2_O, 9:1) compound **13** as a single isomer (539 mg, 0.97 mmol, 68 %) is obtained as a colorless oil.

***R*_f_**=0.37 (n-pentane/Et_2_O 3:1); **[α]_D_^20^**=-2.326° (c=0.430 in DCM); **^1^H NMR** (600 MHz, C_6_D_6_, ppm): δ=7.28‑7.23 (m, 2H), 6.85‑6.80 (m, 2H), 4.39 (dd, *J*=8.4, 3.0 Hz, 1H), 4.37‑4.30 (m, 2H), 4.18 (ddd, *J*=11.0, 9.4, 4.3 Hz, 1H), 4.06 (dd, *J*=6.0, 2.6 Hz, 1H), 3.86 (dt, *J*=10.5, 5.0 Hz, 1H), 3.55 (td, *J*=9.2, 4.7 Hz, 1H), 3.44 (ddd, *J*=9.2, 5.9, 4.4 Hz, 1H), 3.31 (s, 3H), 2.06‑1.99 (m, 1H), 1.82‑1.72 (m, 2H), 1.40 (s, 3H), 1.32 (ddt, *J*=14.1, 6.0, 4.5 Hz, 1H), 1.06 (t, *J*=8.0 Hz, 9H), 0.87 (s, 9H), 0.74 (qd, *J*=7.9, 2.2 Hz, 6H), 0.01 (s, 3H), -0.10 (s, 3H); **^13^C NMR** (151 MHz, C_6_D_6_, ppm): δ=173.5, 159.9, 131.0, 129.8, 128.4, 114.1, 74.0, 73.0, 70.0, 67.2, 64.6, 55.1, 54.8, 34.6, 29.4, 25.9, 18.2, 17.7, 7.4, 6.0, ‑4.2, -4.8; **IR** (neat, cm^-1^): ν^~^=2953 (m), 2933 (m), 2907 (m), 2876 (m), 2857 (m), 1745 (m), 1513 (m), 1463 (m), 1247 (s), 1087 (s), 1034 (s), 1005 (s), 940 (m), 834 (s), 776 (s), 738 (s), 675 (m); **HRMS** (ESI): *m/z* calcd for C_29_H_56_NO_6_Si_2_ [*M*+NH_4_]^+^: 570.3646; found: 570.3638.


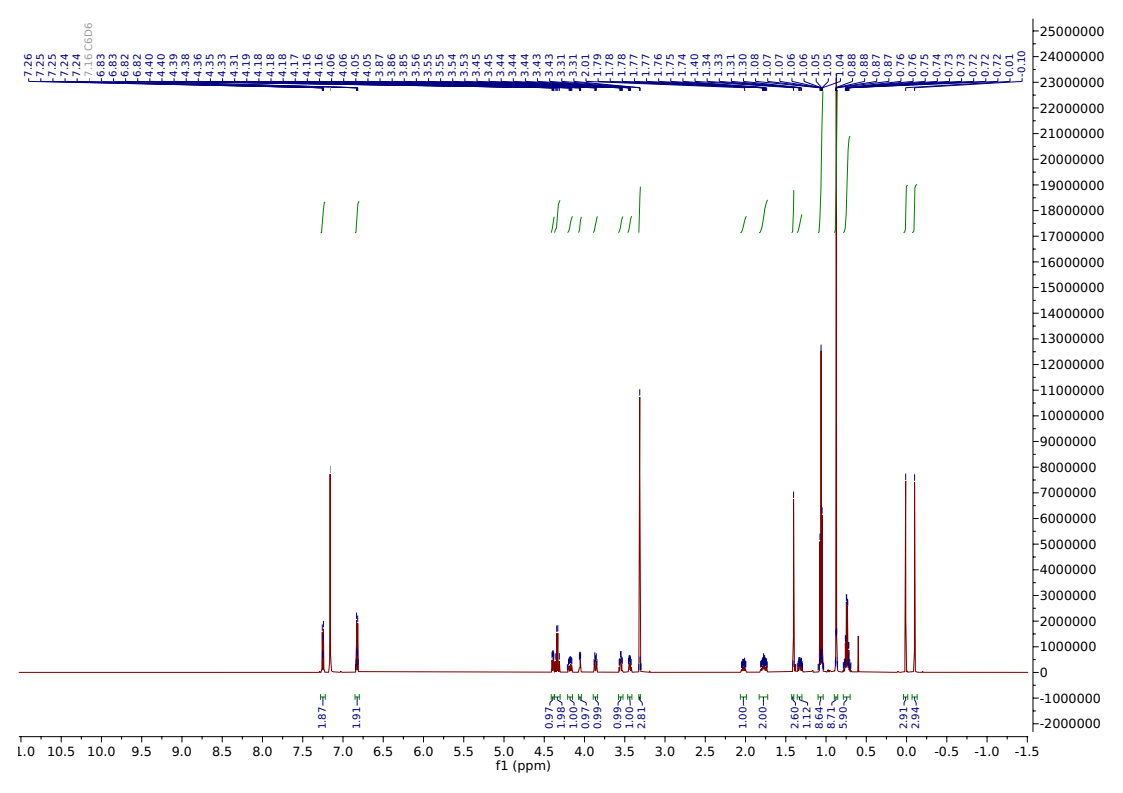

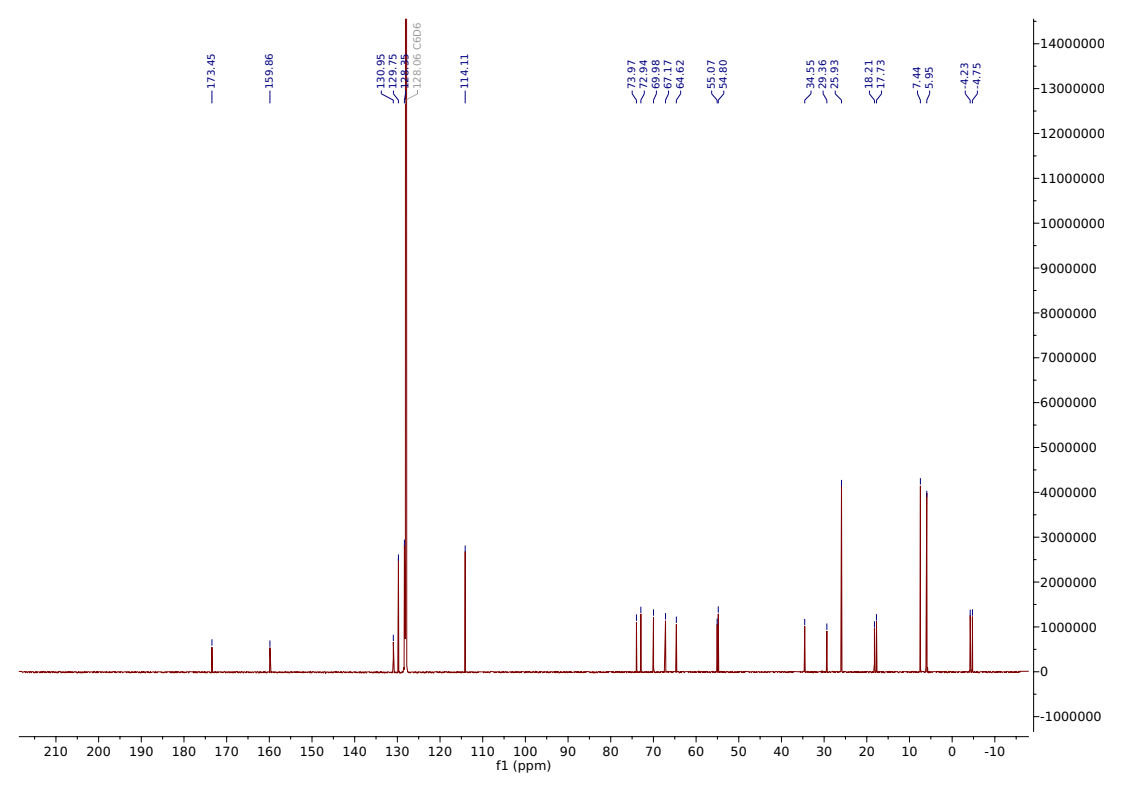

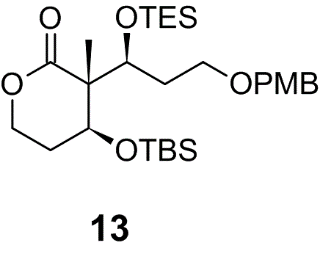


**Methyl (2*R*,3*S*)-3-((*tert*-butyl(dimethyl)silyl)oxy)-5-hydroxy-**

**2-((1*S*)-3-((4-methoxybenzyl)oxy)-1-((triethylsilyl)oxy)propyl)-**

**2-methylpentanoate (14)**


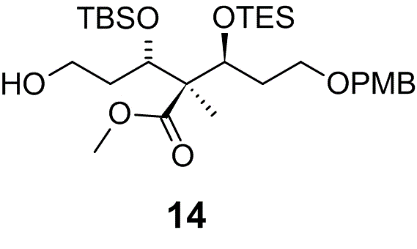


In a 25 ml pointed flask lactone **13** (529 mg, 0.957 mmol, 1.0 eq.) is dissolved in anhydrous THF (0.56 ml) and anhydrous methanol (0.19 ml). A 1.04 M KOH solution in anhydrous methanol (1.26 ml, 1.31 mmol, 1.37 eq.) is added slowly at 0 °C and the reaction solution is allowed to stir for further 50 min at 0 °C. After stirring for 23 h at room temperature the clear bright yellow solution is monitored by TLC (*n*-pentane/Et_2_O, 1:1). Another portion of the same 1.04 M KOH solution (0.2 ml, 0.208 mmol, 0.22 eq.) is injected slowly at room temperature and again the reaction solution is stirred for additional 27 h. A solution of camphorsulfonic acid (351.6 mg, 1.514 mmol, 1.58 eq.) in anhydrous methanol (6 ml) is added slowly dropwise at 0 °C. After stirring for 20 min at 0 °C diethyl ether (15 ml) is added. Then the reaction mixture is allowed to stir further 10 min at 0 °C before it is warmed to room temperature. The reaction solution becomes a white suspension which must be stirred more vigorous to keep it homogeneous. TMSCH_2_N_2_ solution in hexane is injected in several portions until TLC shows full conversion of the carboxylic acid intermediate (R_f_: 0.6 (*n‑*pentane/Et_2_O, 1:1)). Excess of TMSCH_2_N_2_ represented by a yellow suspension must be destroyed by slowly dropwise addition of acetic acid glacial at 0 °C. To the resulting white suspension triethylamine (2.5 ml) is added immediately. After warm up the suspension, dichloromethane (20 ml) and a saturated aqueous NaHCO_3_ solution (4 ml) are poured in a separating funnel. After shaking water (6 ml) is added and the organic layer is separated. The aqueous layer is extracted with dichloromethane (3x20 ml) and the combined organic layers are dried over MgSO_4_, filtered and concentrated under reduced pressure. The residue is purified by column chromatography (*n‑*pentane/ethyl acetate, 95:5 → 9:1) to afford product **14** (390 mg, 0.667 mmol, 70 %) as a colorless oil.

***R*_f_**=0.51 (n-pentane/Et_2_O 1:1); **[α]_D_^20^**=-35.922° (c=0.412 in DCM); **^1^H NMR** (600 MHz, C_6_D_6_, ppm): δ=7.25‑7.21 (m, 2H), 6.83‑6.78 (m, 2H), 4.75 (d, *J*=9.2 Hz, 1H), 4.36‑4.26 (m, 2H), 3.96 (dd, *J*=8.6, 1.8 Hz, 1H), 3.60‑3.53 (m, 3H), 3.49 (s, 3H), 3.40 (ddd, *J*=8.8, 4.9, 3.5 Hz, 1H), 3.31 (s, 3H), 2.34‑2.23 (m, 1H), 1.96‑1.87 (m, 2H), 1.50 (s, 3H), 1.46 (ddt, *J*=14.8, 9.4, 3.4 Hz, 2H), 1.07 (t, *J*=8.0 Hz, 9H), 0.99 (s, 9H), 0.79‑0.63 (m, 6H), 0.17 (s, 3H), 0.12 (s, 3H); **^13^C NMR** (151 MHz, C_6_D_6_, ppm): δ 174.7, 160.0, 130.5, 129.8, 128.4, 114.2, 75.3, 73.2, 72.6, 67.2, 59.3, 57.8, 54.8, 51.0, 36.1, 33.4, 26.3, 18.7, 15.3, 7.4, 6.0, -3.4, -3.9; **IR** (neat, cm^-1^): ν^~^=3786 (w), 3691 (w), 3661 (w), 2951 (m), 2876 (m), 2857 (m), 1724 (m), 1611 (m), 1513 (m), 1460 (m), 1245 (s), 1175 (m), 1074 (s), 1036 (s), 1005 (s), 833 (s), 774 (s), 727 (s), 677 (m); **HRMS** (ESI): *m/z* calcd for C_30_H_57_O_7_Si_2_ [*M*+H]^+^: 585.3643; found: 585.3648.


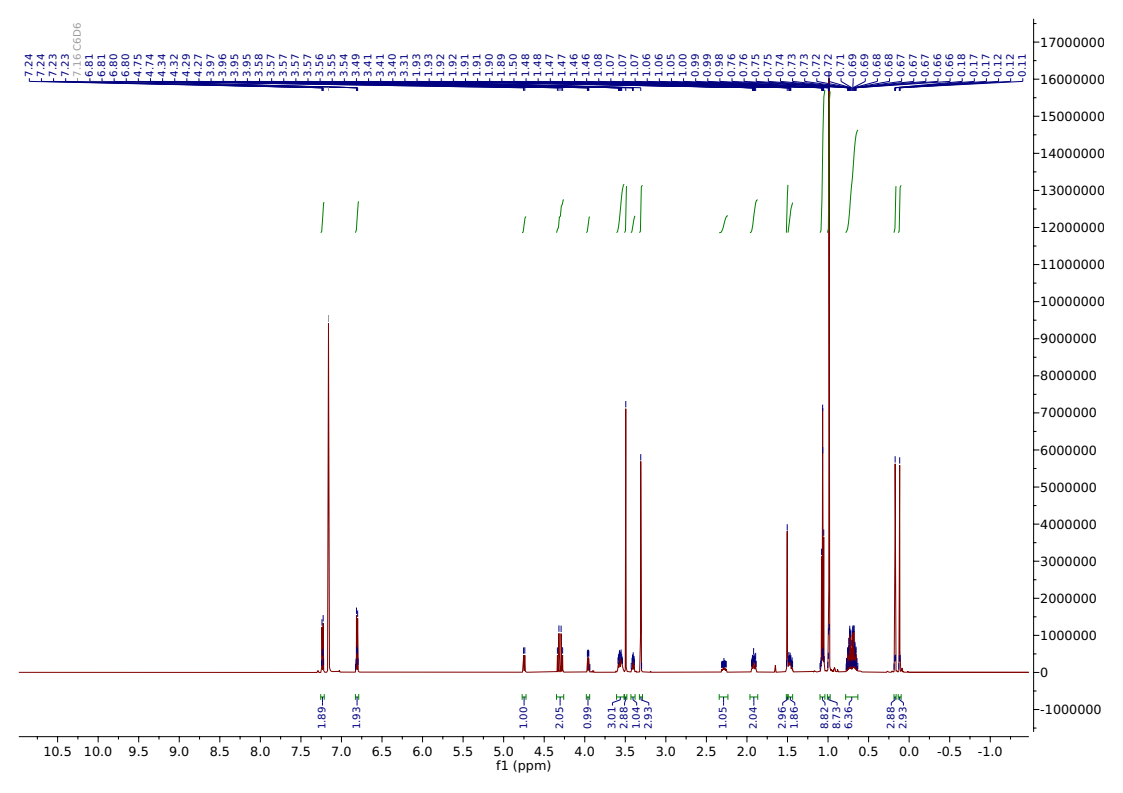

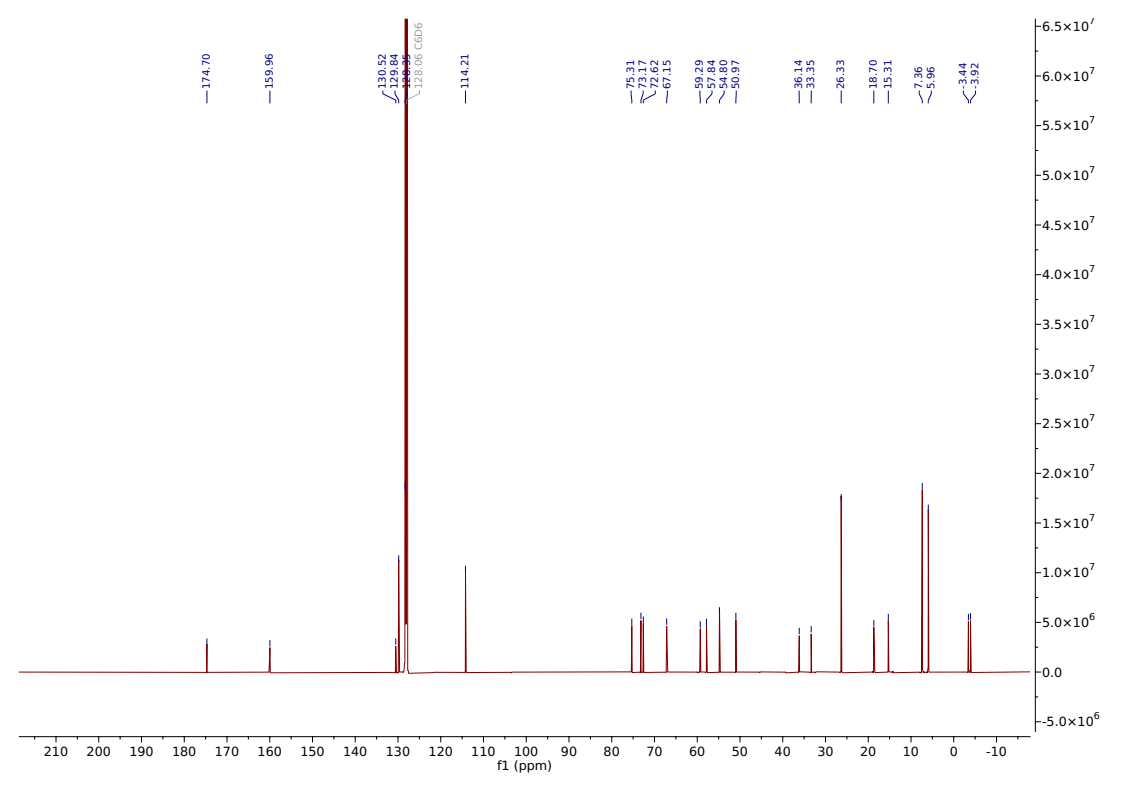

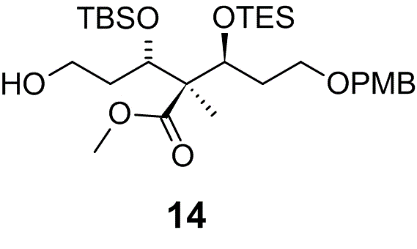


**Methyl (2*R*,3*S*)-3-((*tert*-butyl(dimethyl)silyl)oxy)-**

**2-((1*S*)-3-((4-methoxybenzyl)oxy)-1-((triethylsilyl)oxy)propyl)-**

**2-methyl-5-oxopentanoate (i-15)**


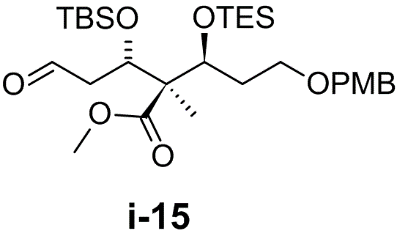


To a solution of the opened lactone **14** (354 mg, 0.605 mmol, 1.0 eq.) in anhydrous dichloromethane (6 ml) NaHCO_3_ (133 mg, 1.574 mmol, 2.6 eq.) and DMP (334 mg, 0.787 mmol, 1.3 eq.) are added sequentially at 0 °C. Before removing of the cooling bath the suspension is allowed to stir for further 15 min at 0 °C. After stirring for 2 h at room temperature the slightly yellow suspension is quenched by addition of saturated aqueous Na_2_S_2_O_3_ solution (5 ml) and saturated aqueous NaHCO_3_ solution (5 ml). The mixture is diluted with diethyl ether (10 ml) and vigorously stirred until two clear layers are visible. After separating of the organic phase the aqueous layer is extracted with diethyl ether (3x10 ml). The combined organic layers are dried over MgSO_4_, filtered, concentrated under reduced pressure and purified by column chromatography (*n‑*pentane/Et_2_O, 6:1) to obtain the aldehyde **i-15** (338 mg, 0.58 mmol, 96 %) as a colorless oil.

***R*_f_**=0.67 (n-pentane/Et_2_O 3:1); **[α]_D_^20^**=-22.374° (c=0.438 in DCM); **^1^H NMR** (600 MHz, C_6_D_6_, ppm): δ=9.44 (d, *J*=1.7 Hz, 1H), 7.23‑7.18 (m, 2H), 6.85‑6.80 (m, 2H), 4.58 (dd, *J*=9.5 Hz, 1.3 Hz, 1H), 4.32‑4.30 (m, 1H), 4.30‑4.23 (m, 2H), 3.50 (ddd, *J*=10.9, 9.2, 3.2 Hz, 1H), 3.47 (s, 3H), 3.31 (s, 3H), 3.29 (ddd, *J*=9.1, 4.9, 3.5 Hz, 1H), 3.03 (dd, *J*=18.5, 2.4 Hz, 1H), 2.81 (ddd, *J*=18.5, 7.4, 1.9 Hz, 1H), 1.59 (dddd, *J*=14.6, 10.8, 4.9, 1.2 Hz, 1H), 1.46 (s, 3H), 1.40 (ddt, *J*=14.6, 9.4, 3.3 Hz, 1H), 1.06 (t, *J*=8.0 Hz, 9H), 0.93 (s, 9H), 0.75‑0.61 (m, 6H), 0.19 (s, 3H), -0.01 (s, 3H); **^13^C NMR** (151 MHz, C_6_D_6_, ppm): δ=200.3, 174.6, 159.9, 130.8, 129.6, 128.4, 114.2, 73.0, 72.24, 72.22, 66.4, 57.4, 54.8, 51.1, 48.3, 33.4, 26.1, 18.5, 15.0, 7.3, 5.9, -3.7, -4.8; **IR** (neat, cm^-1^): ν^~^=2952 (m), 2933 (m), 2876 (m), 2721 (w), 1748 (w), 1722 (s), 1613 (w), 1513 (m), 1463 (m), 1245 (s), 1174 (w), 1081 (s), 1037 (s), 1003 (s), 833 (s), 776 (s), 739 (s), 680 (m); **HRMS** (ESI): *m/z* calcd for C_30_H_55_O_7_Si_2_ [*M*+H]^+^: 583.3486; found: 583.3488.


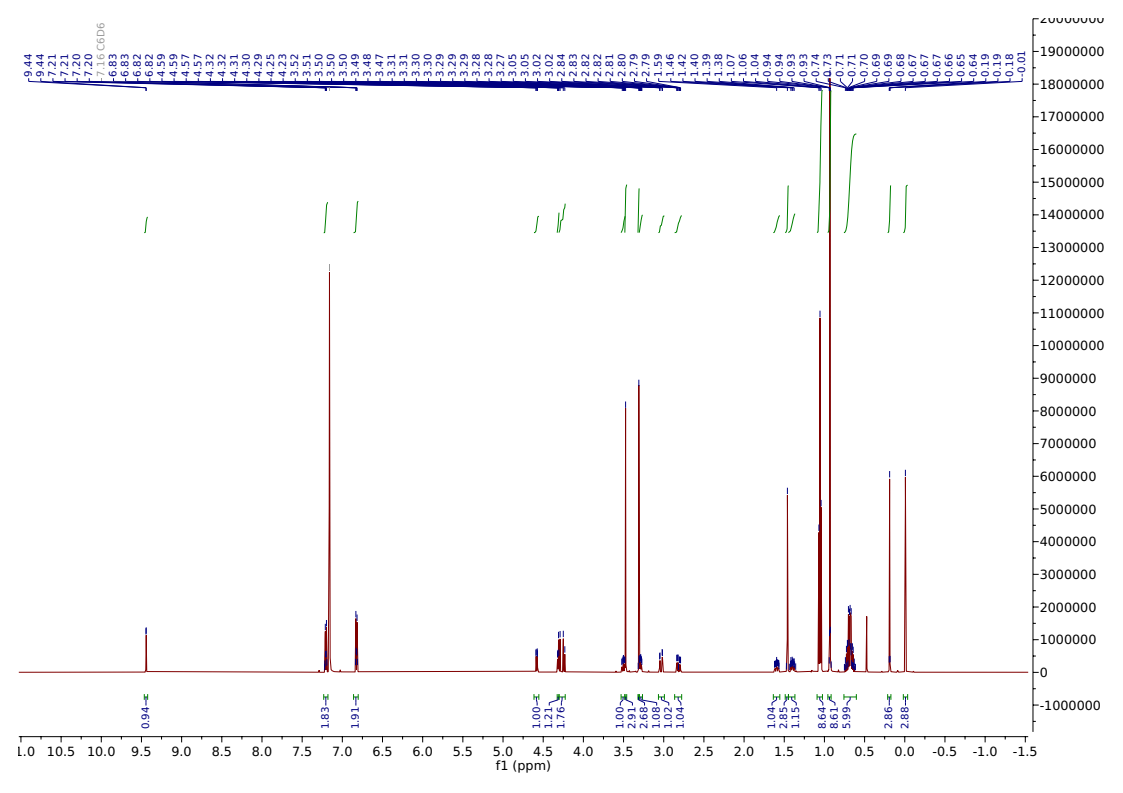

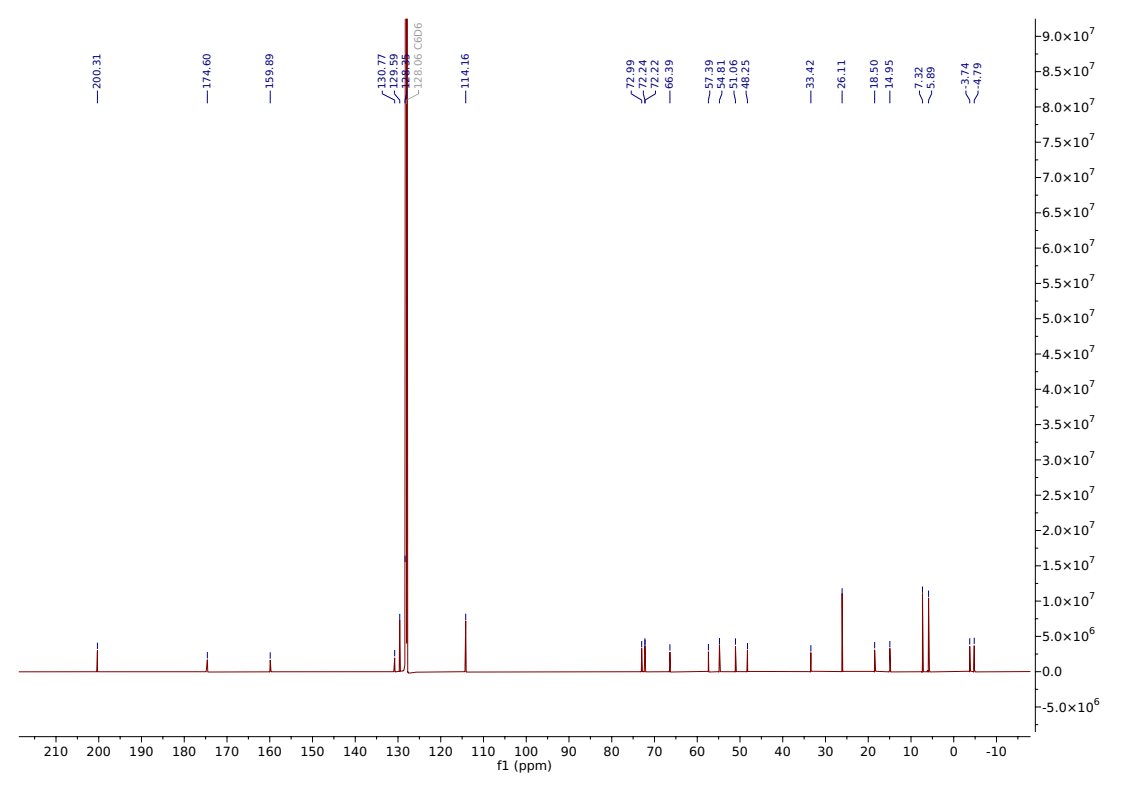

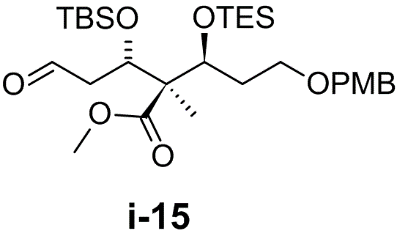


**Methyl (2*S*,3*S*)-3-((*tert*-butyl(dimethyl)silyl)oxy)-**

**2-((1*S*)-3-((4-methoxybenzyl)oxy)-1-((triethylsilyl)oxy)propyl)-**

**2-methylhex-5-enoate (15)**


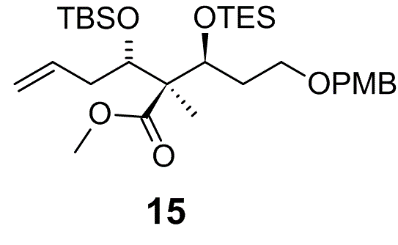


Methyltriphenylphosphonium bromide (239 mg, 0.667 mmol, 1.2 eq.) is suspended in anhydrous THF (1.8 ml) under nitrogen atmosphere. Then 2.5 M *n‑*butyllithium solution in hexane (0.25 ml, 0.625 mmol, 1.12 eq.) is added at 0 °C. After stirring of the red mixture for 1 h at room temperature the orange clear solution is cooled to ‑78 °C. A solution of aldehyde **i-15** (324 mg, 0.556 mmol, 1.0 eq.) in anhydrous THF (3.8 ml) is injected. The reaction mixture is allowed to warm to -30 °C over 1 h before the cooling bath is removed. Then the yellow suspension is stirred for 4.5 h at room temperature. The resulting orange suspension is cooled to ‑20 °C and quenched with saturated aqueous NH_4_Cl solution (5 ml). After removing of the cooling bath the reaction is diluted with diethyl ether (15 ml). The organic phase is separated and the aqueous layer is extracted with diethyl ether (3x15 ml). The combined organic layers are dried over MgSO_4_, filtered and concentrated under reduced pressure. After purification by column chromatography (*n*‑pentane/Et_2_O, 98:2) the desired compound **15** is obtained (273 mg, 0.47 mmol, 85 %) as a colorless oil.

***R*_f_**=0.63 (n-pentane/Et_2_O 9:1); **[α]_D_^20^**=-23.636° (c=0.440 in DCM); **^1^H NMR** (400 MHz; CDCl_3_, ppm): δ=7.29‑7.21 (m, 2H), 6.91‑6.83 (m, 2H), 5.77 (ddt, *J*=17.2, 10.1, 7.1 Hz, 1H), 5.05‑4.93 (m, 2H), 4.48‑4.39 (m, 2H), 4.37 (dd, *J*=9.5, 1.6 Hz, 1H), 3.80 (s, 3H), 3.64 (s, 3H), 3.55 (dd, *J*=8.1, 3.4 Hz, 1H), 3.53‑3.43 (m, 2H), 2.53 (dddt, *J*=14.9, 6.9, 3.0, 1.4 Hz, 1H), 2.34 (dddt, *J*=14.9, 8.2, 7.0, 1.3 Hz, 1H), 1.67 (dtd, *J*=14.0, 8.0, 1.6 Hz, 1H), 1.56 (dddd, *J*=13.9, 9.5, 6.5, 4.4 Hz, 1H), 1.23 (s, 3H), 0.90 (t, *J*=7.9 Hz, 9H), 0.86 (s, 9H), 0.51 (qd, *J*=8.0, 1.6 Hz, 6H), 0.06 (s, 3H), 0.01 (s, 3H); **^13^C NMR** (101 MHz, CDCl_3_, ppm): δ=174.6, 159.3, 137.0, 130.6, 129.4, 116.8, 113.9, 78.3, 72.9, 71.9, 67.2, 58.2, 55.4, 51.2, 37.8, 33.2, 26.1, 18.4, 14.9, 7.1, 5.6, -2.9, -3.9; **IR** (neat, cm^-1^): ν^~^=2951 (m), 2934 (m), 2876 (m), 2857 (m), 1724 (m), 1613 (w), 1513 (m), 1462 (m), 1245 (s), 1075 (s), 1037 (m), 1004 (m), 913 (m), 826 (m), 810 (m), 774 (m), 729 (s); **HRMS** (ESI): *m/z* calcd for C_31_H_57_O_6_Si_2_ [*M*+H]^+^: 581.3694; found: 581.3708.


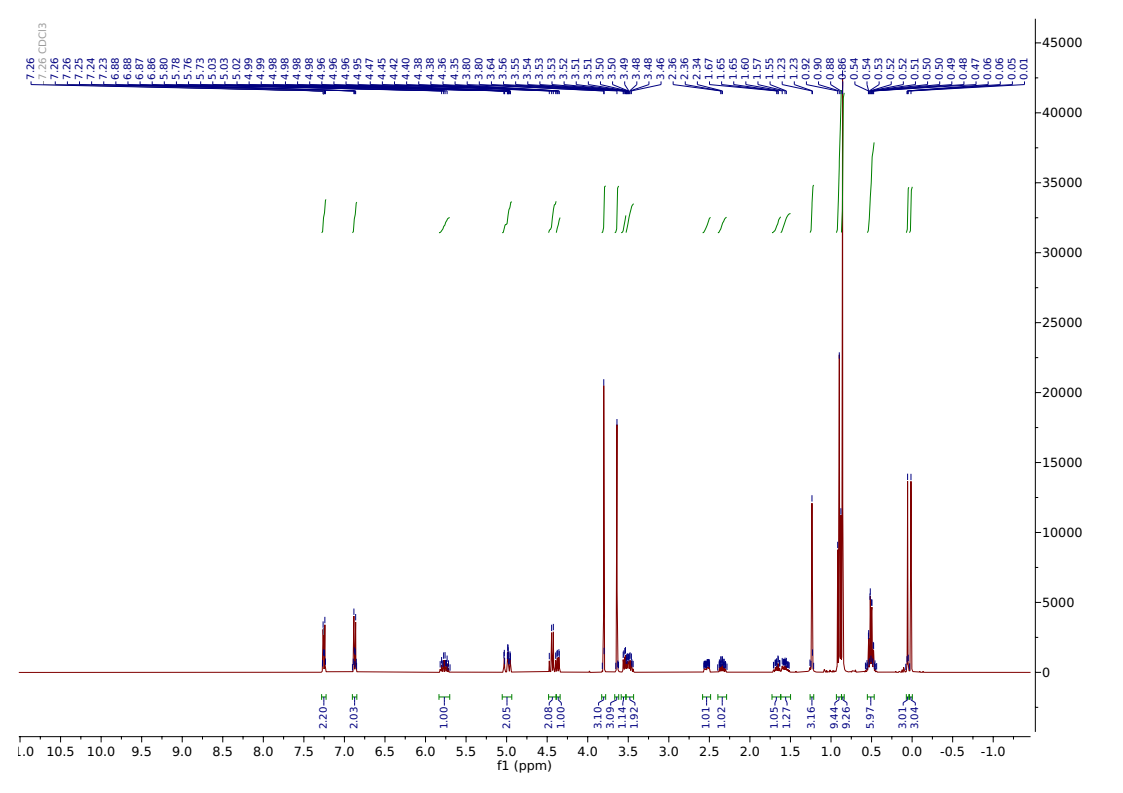

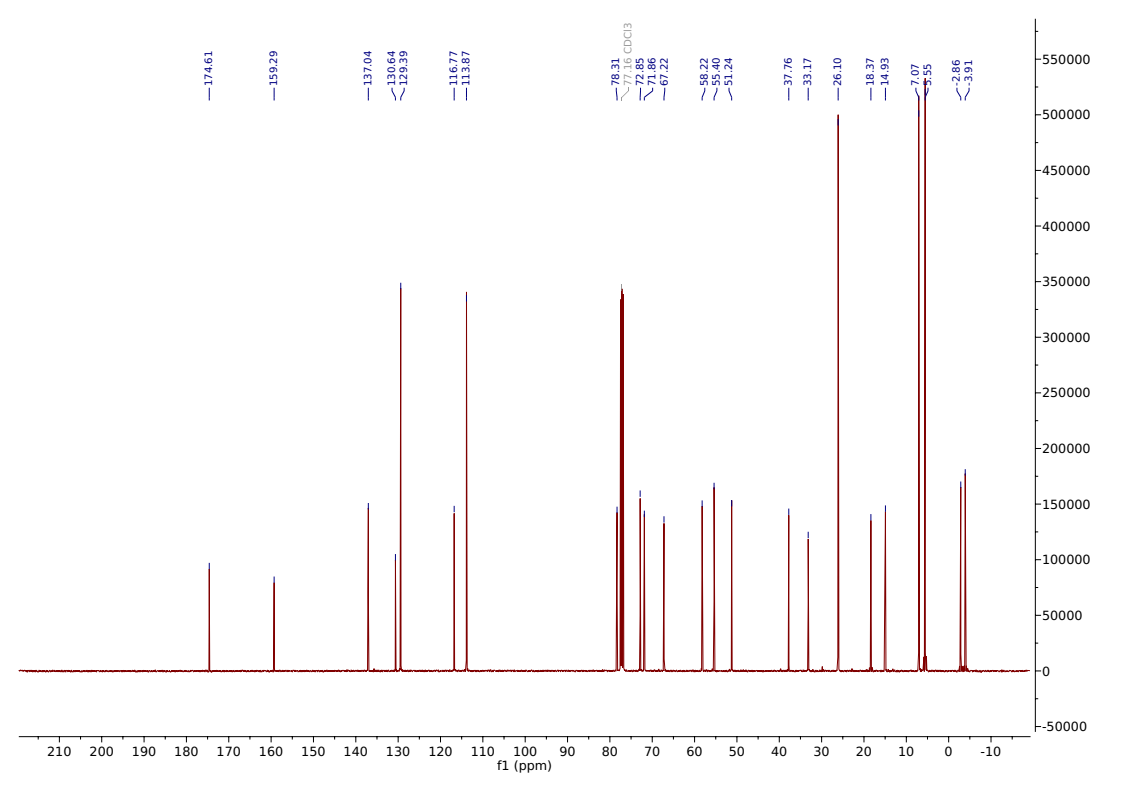

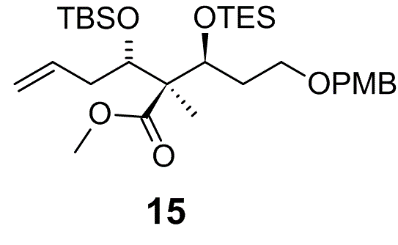


**Methyl (2*R*,3*S*,4*E*)-3-((*tert*-butyl(dimethyl)silyl)oxy)-**

**2-((1*S*)-3-((4-methoxybenzyl)oxy)-1-((triethylsilyl)oxy)propyl)-**

**2-methylhex-4-enoate (16)**


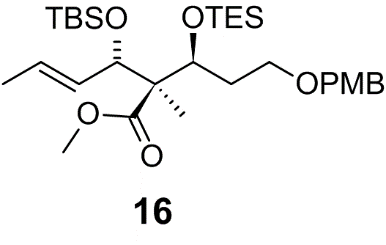


The terminal alkene **15** (268 mg, 0.461 mmol, 1.0 eq.) is dissolved in methanol (6.1 ml, 99.5 % laboratory grade). After sequential addition of triethylamine (0.07 ml, 0.461 mmol, 1.0 eq.) and Grubbs II M204 (40.9 mg, 0.048 mmol, 0.1 eq.) the flask is closed by a glass cap and heated to 55 °C (oil bath temperature). The yellow/brown solution is stirred for 18 h after which TLC monitoring (*n*‑pentane/Et_2_O, 9:1) indicates full conversion. The solvent is removed under reduced pressure and the brown residue is purified by column chromatography (*n*‑pentane/Et_2_O, 99:1 → 98:2) to afford the final product **16** (242 mg, 0.417 mmol, 90 %, E/Z ratio 12:1) as a colorless oil.

***R*_f_**=0.57 (n-pentane/Et_2_O 9:1); **[α]_D_^20^**=-16.000° (c=0.250 in CHCl_3_); **^1^H NMR** (400 MHz, CDCl_3_, ppm): δ=7.29‑7.21 (m, 2H), 6.91‑6.84 (m, 2H), 5.67 (ddq, *J*=15.5, 9.1, 1.6 Hz, 1H), 5.46 (dq, *J*=15.5, 6.4 Hz, 1H), 4.47‑4.35 (m, 2H), 4.11 (dd, *J*=9.4, 1.5 Hz, 1H), 3.84 (d, *J*=9.0 Hz, 1H), 3.80 (s, 3H), 3.62 (s, 3H), 3.49 (td, *J*=8.9, 4.4 Hz, 1H), 3.37 (dt, *J*=8.8, 7.7 Hz, 1H), 1.79 (dddd, *J*=13.7, 9.0, 7.5, 1.5 Hz, 1H), 1.61 (dd, *J*=6.4, 1.6 Hz, 3H), 1.53 (ddd, *J*=9.4, 8.0, 4.2 Hz, 1H), 1.22 (s, 3H), 0.89 (t, *J*=7.9 Hz, 9H), 0.83 (s, 9H), 0.56‑0.42 (m, 6H), -0.01 (s, 3H), -0.08 (s, 3H); **^13^C NMR** (101 MHz, CDCl_3_, ppm): δ=174.8, 159.3, 130.9, 130.8, 129.4, 128.9, 113.9, 79.6, 72.7, 72.3, 67.8, 58.4, 55.4, 51.2, 32.9, 25.8, 18.1, 17.7, 14.1, 7.1, 5.6, -3.4, -4.9; **IR** (neat, cm^-1^): ν^~^=2950 (m), 2876 (m), 2856 (m), 1725 (m), 1613 (w), 1513 (m), 1461 (w), 1246 (s), 1087 (s), 1038 (s), 1005 (s), 974 (m), 834 (s), 775 (s), 735 (s), 677 (w); **HRMS** (ESI): *m/z* calcd for C_31_H_57_O_6_Si_2_ [*M*+H]^+^: 581.3694; found: 581.3708.


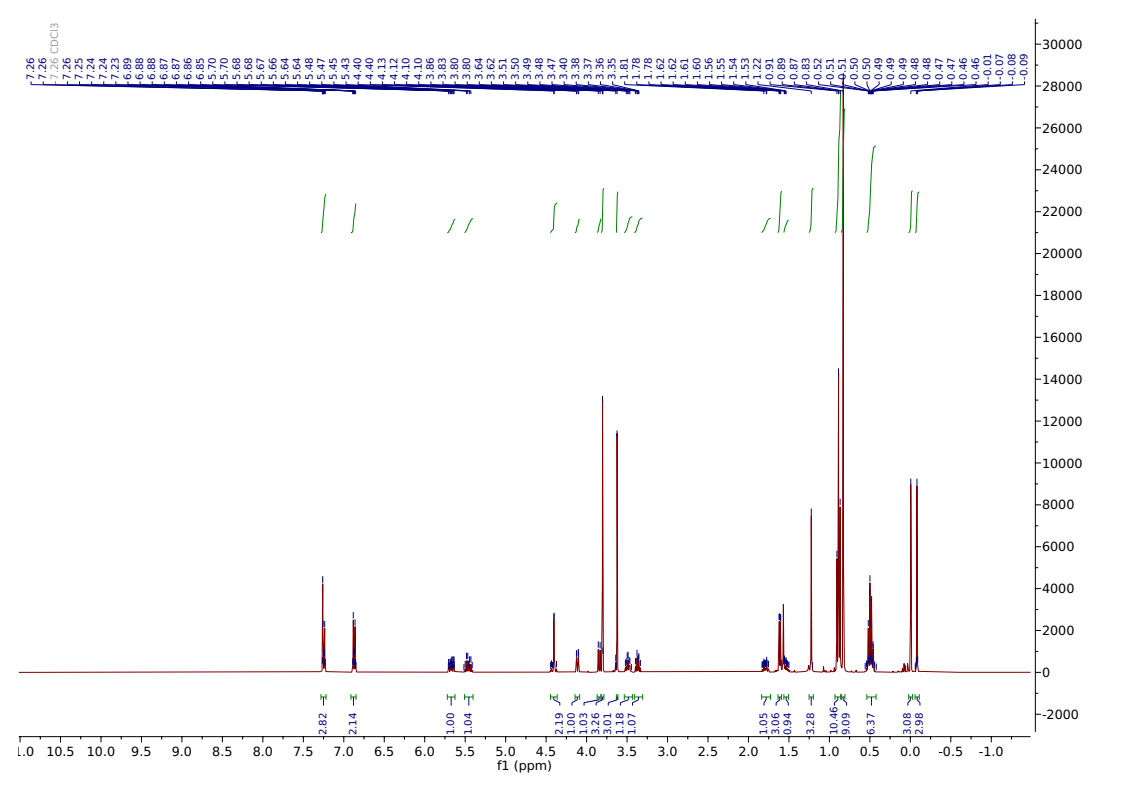

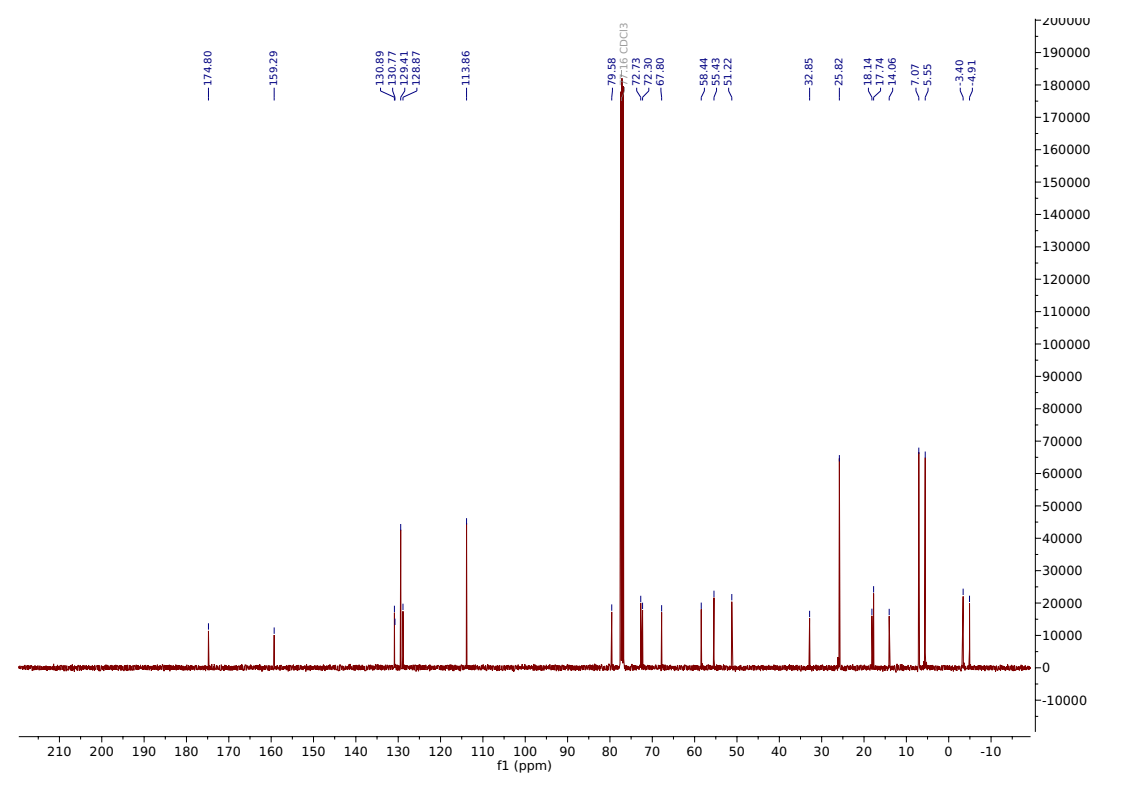

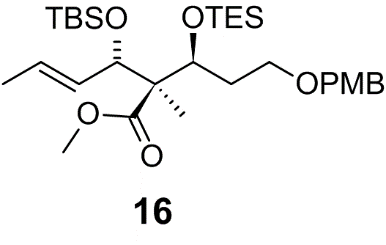


**Methyl (2*R*,3*S*,4*E*)-3-((*tert*-butyl(dimethyl)silyl)oxy)-2-methyl-**

**2-((1*S*)-3-oxo-1-((triethylsilyl)oxy)propyl)hex-4-enoate (17)**


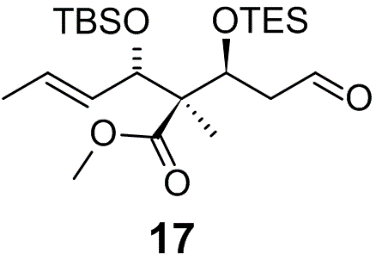


Compound **16** (160 mg, 0.2754 mmol, 1.0 eq.) is dissolved in dichloromethane (5.80 ml). Then an aqueous pH 7 phosphate buffer solution (0.58 ml) is poured in. After that DDQ (93.8 mg, 0.4131 mmol, 1.5 eq.) is added and the mixture is stirred for 1 h at room temperature. The reaction is quenched with saturated aqueous NaHCO_3_ solution (15 ml) and diluted with dichloromethane (15 ml). The organic layer is separated and the aqueous layer is extracted with dichloromethane (3x15 ml). The combined organic layers are dried over MgSO_4_, filtered and concentrated under reduced pressure. This crude mixture, which is directly used for the next step, is dissolved in anhydrous dichloromethane (2.8 ml) and cooled to 0 °C. After sequential addition of NaHCO_3_ (60.15 mg, 0.716 mmol, 2.6 eq.) and DMP (151.85 mg, 0.358 mmol, 1.3 eq.) the resulting suspension is stirred for 15 min at 0 °C. Then stirring is continued for 3 h at room temperature. The reaction is quenched by addition of saturated aqueous Na_2_S_2_O_3_ solution (3 ml) and saturated aqueous NaHCO_3_ solution (3 ml) and the mixture is diluted with diethyl ether (15 ml). The organic layer is separated and the aqueous layer is extracted with diethyl ether (3x20 ml). The combined organic layers are dried over MgSO_4_, filtered, concentrated under reduced pressure and purified by column chromatography (*n‑*pentane/Et_2_O, 95:5) to obtain the aldehyde **17** (87 mg, 0.19 mmol, 69 %) as a colorless oil.

***R*_f_**=0.6 (n-pentane/Et_2_O 9:1); **[α]_D_^20^**=+6.923° (c=0.26 in CHCl_3_); **^1^H NMR** (600 MHz, C_6_D_6_, ppm): δ=9.62 (dd, *J*=2.4, 0.9 Hz, 1H), 5.84 (ddq, *J*=15.5, 9.2, 1.7 Hz, 1H), 5.18 (dqd, *J*=15.5, 6.5, 0.7 Hz, 1H), 4.92 (dd, *J*=7.9, 2.5 Hz, 1H), 3.87 (dd, *J*=9.2, 0.7 Hz, 1H), 3.46 (s, 3H), 2.45 (ddd, *J*=17.3, 2.6, 0.9 Hz, 1H), 2.39 (ddd, *J*=17.3, 7.9, 2.4 Hz, 1H), 1.46 (dd, *J*=6.5, 1.7 Hz, 3H), 1.38 (s, 3H), 1.01 (t, *J*=8.0 Hz, 9H), 0.95 (s, 9H), 0.67 (qd, *J*=7.9, 2.1 Hz, 6H), 0.03 (s, 3H), 0.00 (s, 3H); **^13^C NMR** (151 MHz, C_6_D_6_, ppm): δ=199.8, 174.0, 131.3, 129.3, 79.8, 70.3, 58.0, 51.2, 48.3, 26.3, 26.0, 18.3, 17.5, 14.3, 7.21, 7.20, 5.64, 5.61, -3.3, -4.7; **IR** (neat, cm^-1^): ν^~^=2953 (m), 2931 (m), 2878 (m), 2858 (m), 1728 (s), 1241 (m), 1087 (s), 1052 (s), 1004 (m), 977 (m), 856 (m), 835 (s), 775 (s), 731 (s); **HRMS** (ESI): *m/z* calcd for C_23_H_47_O_5_Si_2_ [*M*+H]^+^: 459.2962; found: 459.2961.


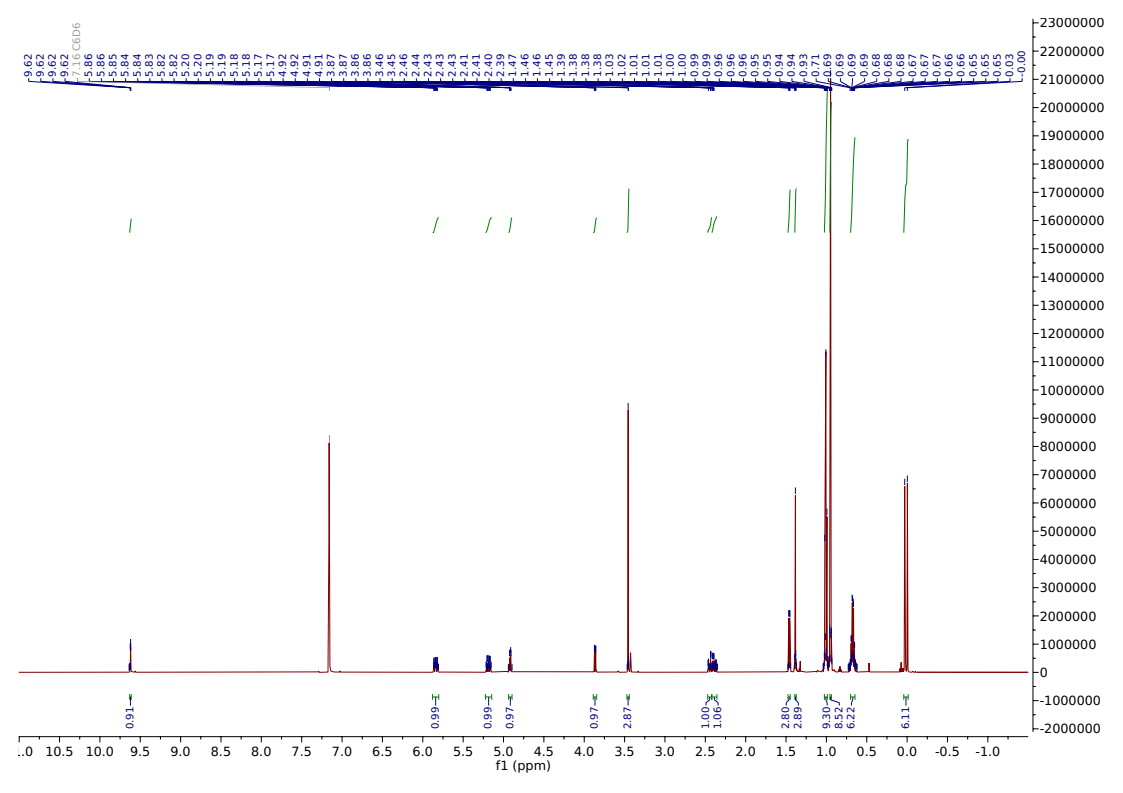

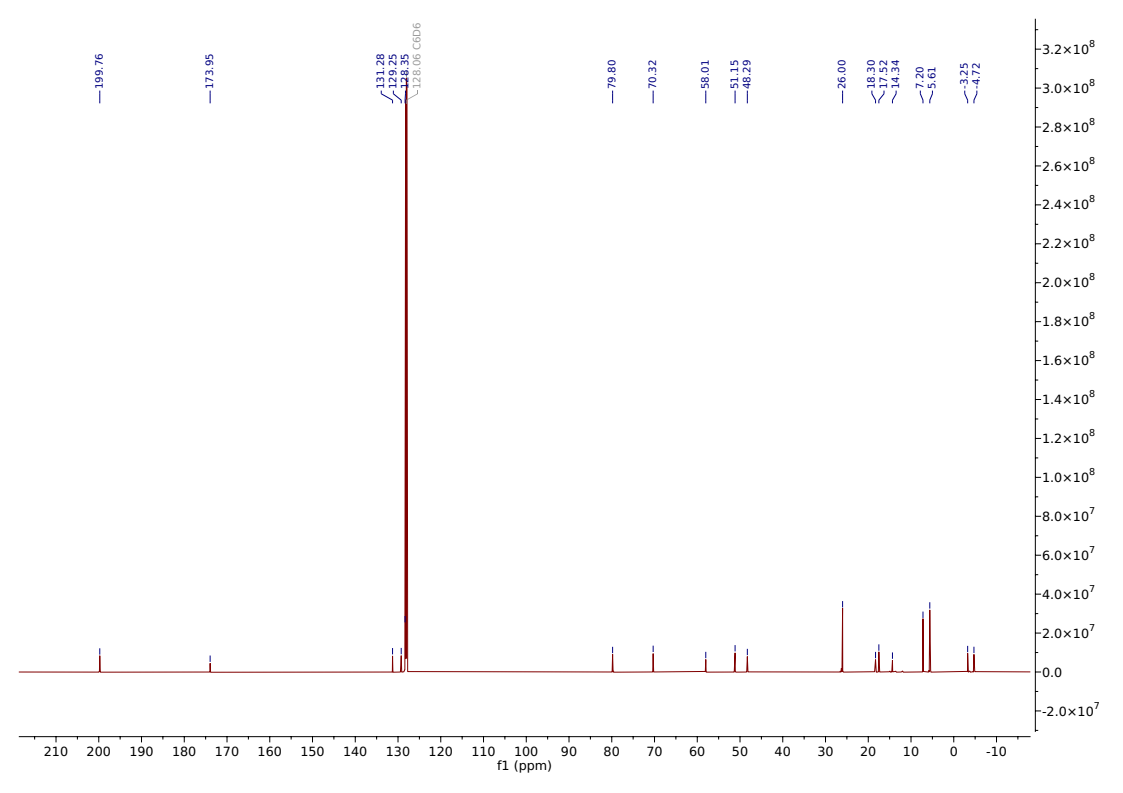

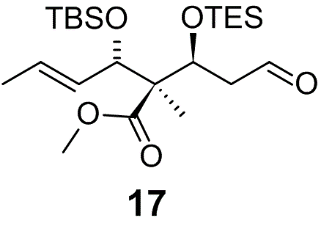


**Methyl (2*R*,3*S*,4*E*)-3-((*tert*-butyl(dimethyl)silyl)oxy)-**

**2-((1*S*,3*Z*)-1-hydroxy-4-iodobut-3-en-1-yl)-2-methylhex-4-enoate (7)**


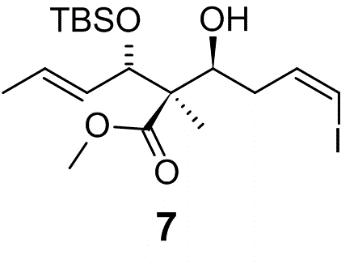


(Iodomethyl)triphenylphosphonium iodide (115.6 mg, 0.218 mmol, 1.25 eq.) is suspended in anhydrous THF (1.25 ml). Then 2M NaHMDS solution in THF (0.12 ml, 0.227 mmol, 1.3 eq.) is added to the suspension at room temperature and the mixture is stirred for 10 min. The resulting orange solution is cooled to -78 °C. Aldehyde **17** (80 mg, 0.1744 mmol, 1.0 eq.) is dissolved in anhydrous THF (2.5 ml) and the mixture is injected drop wise. After stirring for 25 min at -78 °C the reaction mixture is allowed to warm up to room temperature over 1h. Then stirring is continued for additional 3.5 h. The reaction is stopped by filtration through a pad of silica, which is flushed with diethyl ether (200 ml). After evaporation of the solvent the crude mixture is purified by column chromatography (*n‑*pentane/Et_2_O, 99:1) to obtain an inseparable mixture (57 mg) of product (R_f_: 0.9 (*n‑*pentane/Et_2_O 9:1)) and an unknown compound.

This mixture (57 mg, 0.098 mmol, 1.0 eq.) is used for the next step as if it would be the pure substance. It is dissolved in anhydrous methanol (1.0 ml) and pyridinium *p‑*toluenesulfonate (27.6 mg, 0.11 mmol, 1.1 eq.) is added. The solution is stirred for 20 h at 0 °C. After evaporation of the solvent, the residue is purified by column chromatography (*n‑*pentane/Et_2_O, 9:1) to obtain the vinyl iodide **7** (27 mg, 0.058 mmol, 33 %, *E*/*Z* = 1:10) as a colorless oil.

***R*_f_**=0.15 (n-pentane/Et_2_O 9:1); **[α]_D_^25^**=-37.500° (c=0.2 in CHCl_3_); **^1^H NMR** (400 MHz, C_6_D_6_, ppm): δ=6.25 (td, *J*=7.1, 6.2 Hz, 1H), 5.96 (dt, *J*=7.3, 1.4 Hz, 1H), 5.57 – 5.44 (m, 2H), 4.85 – 4.78 (m, 1H), 3.90 (td, *J*=10.3, 2.7 Hz, 1H), 3.24 (s, 3H), 3.22 (s, 1H), 2.51 (dddd, *J*=14.8, 7.0, 2.7, 1.5 Hz, 1H), 2.16 (dddd, *J*=14.8, 10.5, 6.2, 1.5 Hz, 1H), 1.47 – 1.45 (m, 3H), 1.25 (s, 3H), 1.00 (s, 9H), 0.21 (s, 3H), 0.10 (s, 3H); **^13^C NMR** (101 MHz, C_6_D_6_, ppm): δ=175.3, 139.3, 131.1, 128.7, 83.7, 75.9, 73.4, 56.8, 51.3, 39.2, 26.1, 18.4, 17.7, 14.1, ‑3.8, -4.8; **IR** (neat, cm^-1^): ν^~^=3500 (w), 2952 (m), 2929 (m), 2856 (m), 1728 (m), 1702 (m), 1463 (m), 1435 (m), 1251 (s), 1088 (s), 1050 (s), 836 (s), 775 (s); **HRMS** (ESI): *m/z* calcd for C_18_H_37_NIO_4_Si [*M*+NH_4_]^+^: 486.1537; found: 486.1539.


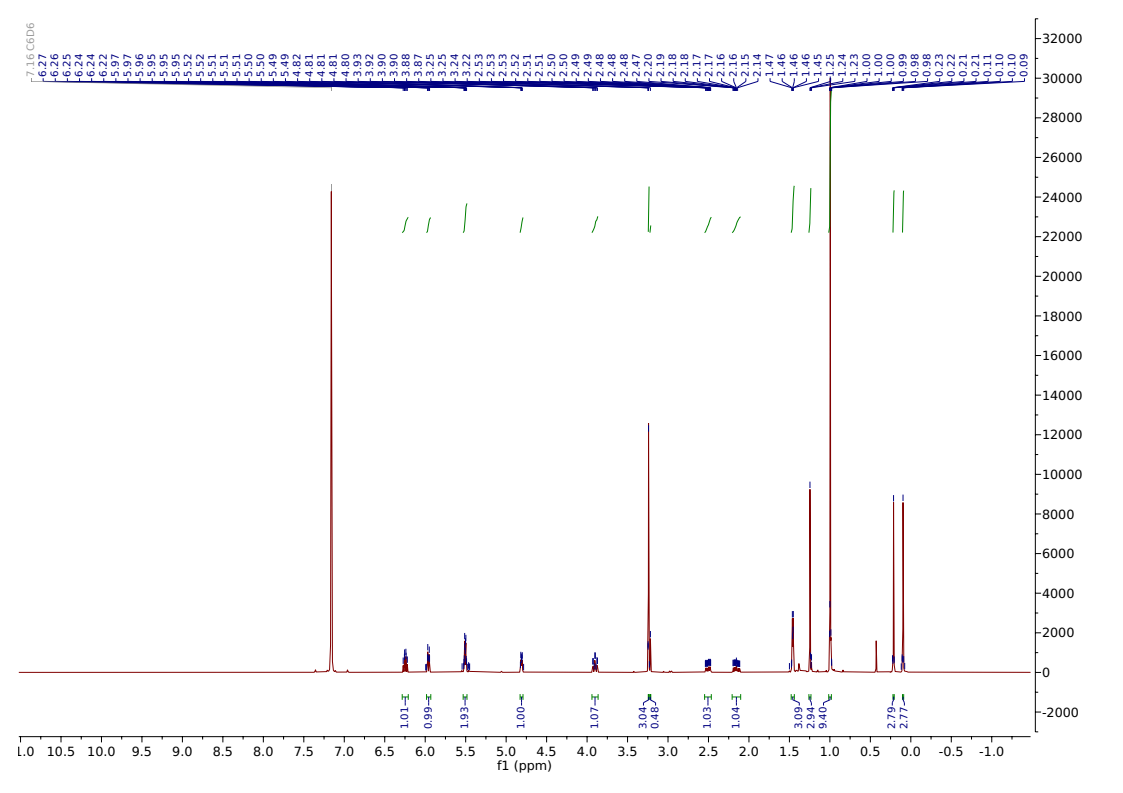

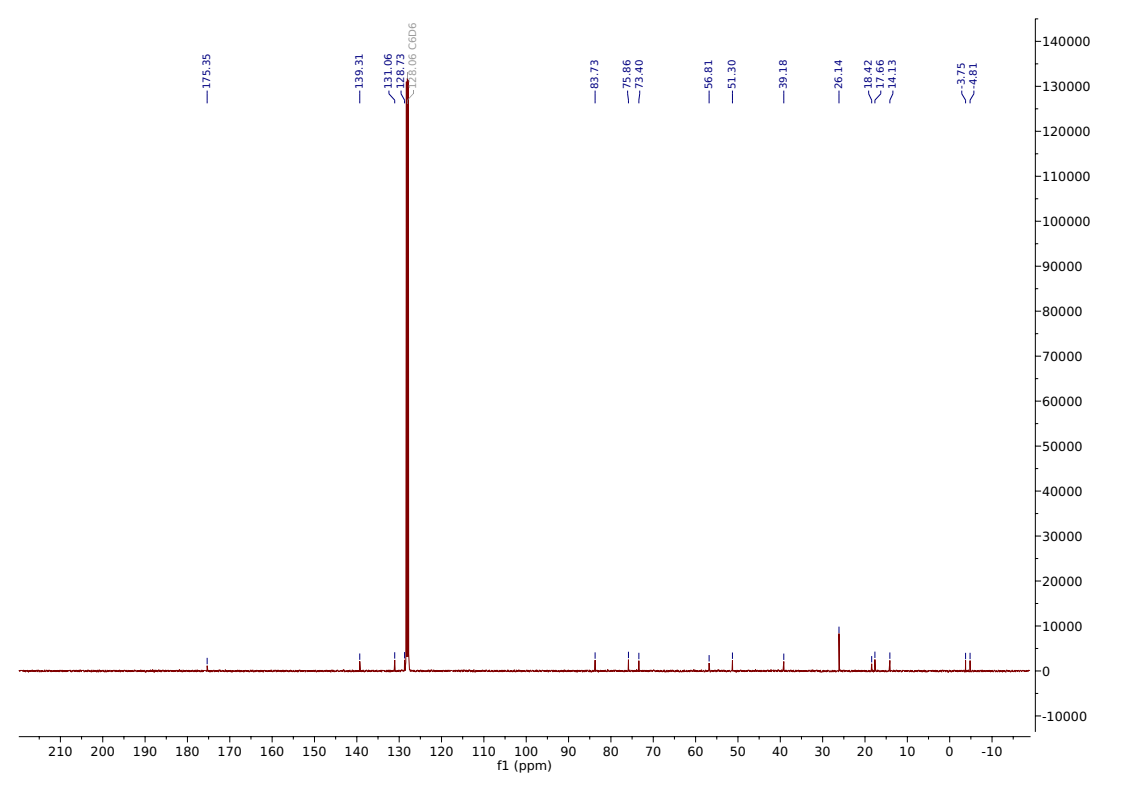

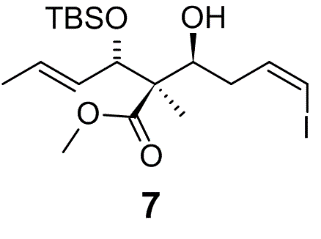


**3-((4-methoxybenzyl)oxy)propan-1-ol (i-12)**


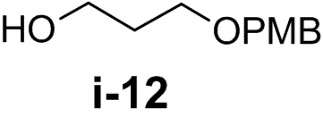


1,3-propandiol (1.65 g, 21.7 mmol, 1.0 eq.) is dissolved in anhydrous dichloromethane (13 ml). Then *p*-methoxybenzyl alcohole (3.0 ml, 24.06 mmol, 1.1 eq.) and Amberlyst 15 (0.165 g, 0.1 eq. (w/w)) are added sequentially. After refluxing for 6.5 h the reaction mixture is stirred over night at room temperature. Then the solution is refluxed again for 6.5 h. After that MgSO_4_ is added at room temperature. MgSO_4_ and Amberlyst 15 are removed by filtration. The residue in the filter is washed with dichloromethane. After concentration under reduced pressure the crude is purified by column chromatography (*n‑*pentane/Et_2_O, 1:2) to afford product **i-12** (3.131 g, 15.95 mmol, 73.5 %) as a colorless oil (see ref. [24]).

***R*_f_**=0.18 (*n*-pentane/Et_2_O, 1:2); **^1^H NMR** (400 MHz, CDCl_3_, ppm): δ=7.27 – 7.20 (m, 2H), 6.90 – 6.83 (m, 2H), 4.43 (s, 2H), 3.78 (s, 3H), 3.73 (t, *J*=5.8 Hz, 2H), 3.60 (t, *J*=5.9 Hz, 2H), 2.64 (s, 1H), 1.83 (p, *J*=5.9 Hz, 2H); **^13^C NMR** (101 MHz, CDCl_3_, ppm): δ=159.2, 130.2, 129.3, 113.8, 72.9, 68.8, 61.5, 55.3, 32.2; **IR** (neat, cm^-1^): ν^~^=3747 (w), 3395 (w br), 2936 (m), 2860 (m), 1612 (s), 1512 (s), 1244 (s), 1174 (s), 1081 (s), 1031 (s), 817 (s), 514 (s).


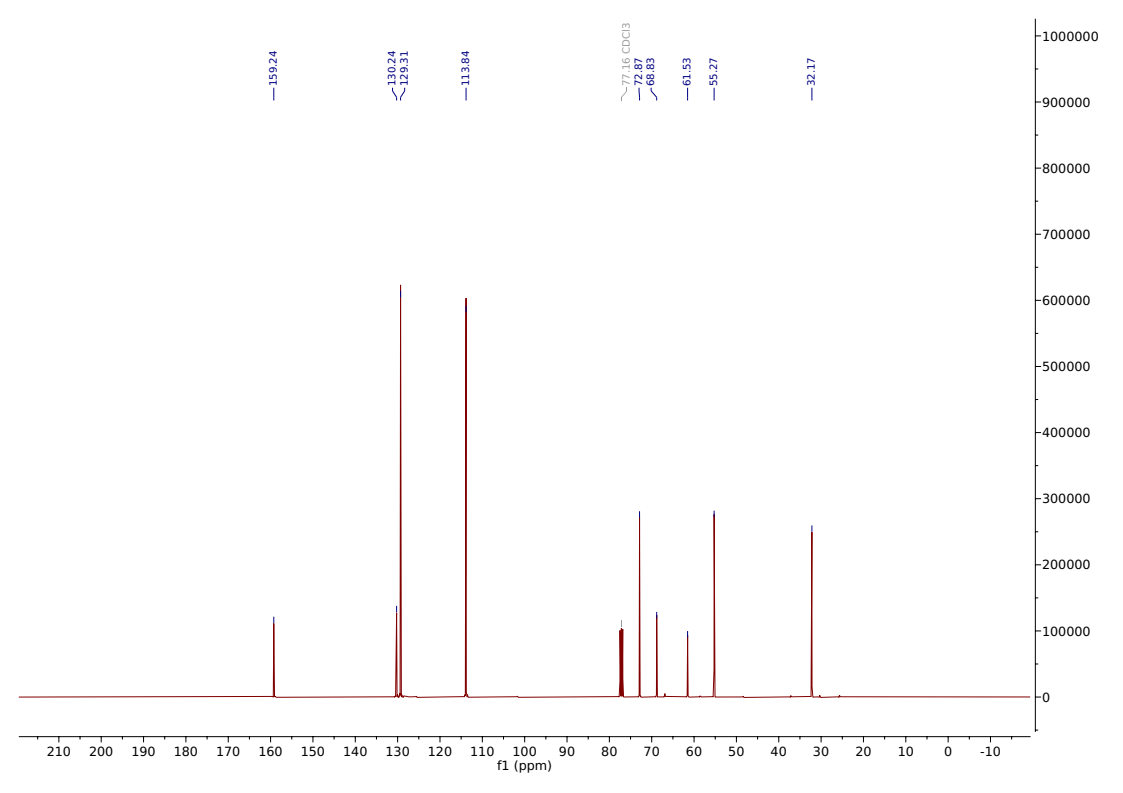

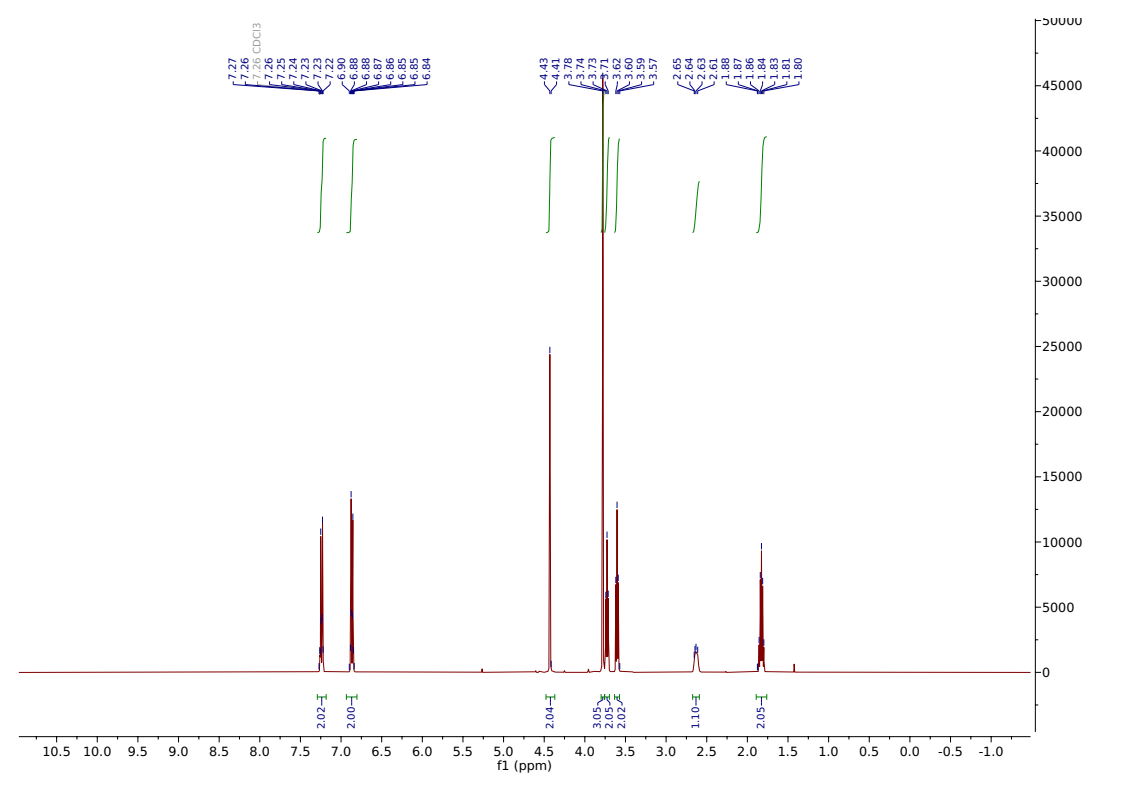

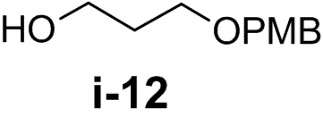


**3-((4-methoxybenzyl)oxy)propanal (12)**


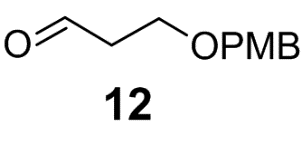


Compound **i-12** (3.04 g, 15.5 mmol, 1.0 eq) is dissolved in anhydrous dichloromethane (59 ml). After sequential addition of DMSO (12 ml) and triethylamine (11 ml, 77.5 mmol, 5.0 eq) the solution is cooled to 0 °C. Then sulphur trioxide pyridine complex (9.87 g, 62 mmol, 4.0 eq.) is slowly added in portions. The solution is stirred for 3 h at 0 °C. After that diethyl ether (250 ml) is added and the organic phase is washed at first with water (2x70 ml) and then with an aqueous saturated CuSO_4_ solution (70 ml). After washing several times with more water to remove the CuSO_4_ solution residues, the organic layer is treated with brine (70 ml), dried over MgSO_4_ and filtered. The solvent is removed under reduced pressure and the residue is purified by column chromatography (*n*-pentane/Et_2_O, 2:1) to obtain the protected aldehyde **12** (2.22 g, 11.43 mmol, 74 %) as a colorless oil.

***R*_f_**=0.57 (*n*-pentane/Et_2_O, 1:2); **^1^H NMR** (400 MHz, CDCl_3_, ppm): δ=9.76 (t, *J*=1.8 Hz, 1H), 7.28 – 7.20 (m, 2H), 6.91 – 6.83 (m, 2H), 4.45 (s, 2H), 3.79 (s, 3H), 3.76 (t, *J*=6.1 Hz, 2H), 2.66 (td, *J*=6.1, 1.9 Hz, 2H); **^13^C NMR** (101 MHz, CDCl_3_, ppm): δ=201.3, 159.4, 130.0, 129.4, 113.9, 72.9, 63.6, 55.3, 43.9; **IR** (neat, cm^-1^): ν^~^=2933 (w), 2860 (m), 2837 (m), 2732 (w), 1722 (s), 1612 (s), 1512 (s), 1244 (s), 1174 (s), 1088 (s), 1031 (s), 817 (s).

Further analytical data see ref. [25].


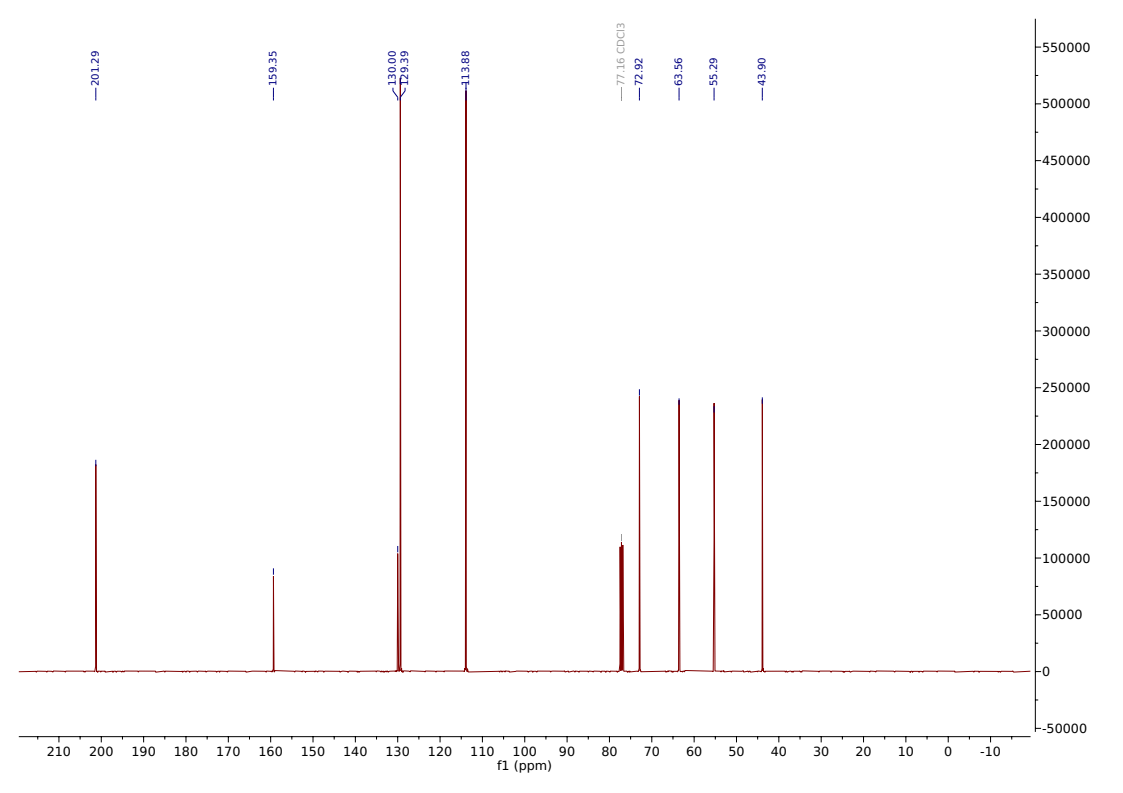

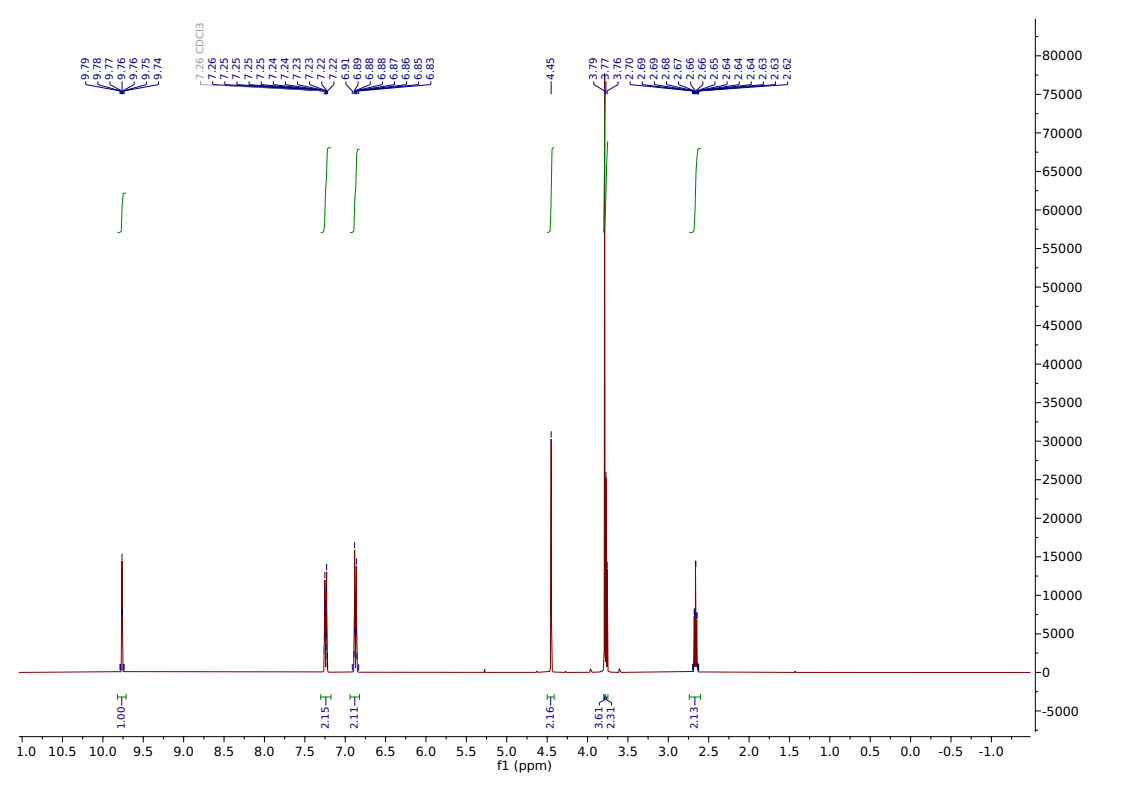

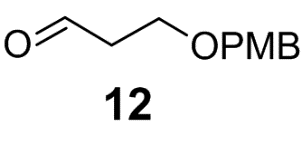


Scheme S3: Synthesis of fragment **6**

**(Iodoethynyl)trimethylsilane (SA-1)^[15][21][22]^**

To a solution of trimethylsilylacetylene (10 g, 87.55 mmol, 1.0 equiv) in dry THF (150 mL), n-BuLi (2.5 M in hexane, 70 mL, 175.10 mmol, 2.0 equiv) was added dropwise at -78 °C, and the mixture was stirred for 60 minutes. A solution of I₂ (26.5 g, 105.06 mmol, 1.2 equiv) in dry THF (80 mL) was then added dropwise. The reaction mixture was stirred for an additional 15 minutes at -78 °C before quenching with saturated aqueous Na₂S₂O₃ (200 mL). Et₂O (100 mL) and water (100 mL) were added, and the layers were separated. The aqueous layer was extracted with Et₂O (3×100 mL). The combined organic layers were dried over MgSO₄, filtered, and concentrated under reduced pressure (100 mbar, 40 °C) to afford **SA-1** (13.34 g, 59.53 mmol, 68%) as a pale-yellow liquid with a fruity odor. The product was used directly in the next reaction without purification.

**General data:** C_5_H_9_ISi; FW: 224.114 ; **TLC**: R_f_=0.54 (Pentane); UV (+); KMnO_4_

**^1^H-NMR** (600 MHz, CDCl_3_) δ 0.18 (s, 9H).

**^13^C-NMR** (151 MHz, CDCl_3_) δ 104.37, 30.47, 0.04. (in agreement with lit.[26]).

**5-(Trimethylsilyl)penta-2,4-diyn-1-ol (SA-2)^[15][23]^**

A degassed solution of dry THF (300 mL) and DIPA (30 mL, 22.52 g, 150.0 mmol, 3.0 equiv) was prepared by three freeze-pump-thaw cycles. To this solution, Pd(PPh₃)₂Cl₂ (676 mg, 1.17 mmol, 2 mol%) and CuI (223 mg, 1.17 mmol, 2 mol%) were added, followed by **SA-1** (13.00 g, 58.03 mmol, 1.0 equiv) and propargyl alcohol (3.90 g, 43.44 mmol, 1.2 equiv). The resulting yellow-brown solution was stirred at room temperature for 2 h 30 min. The reaction mixture was filtered over a pad of Celite and washed with Et₂O. The filtrate was concentrated under reduced pressure to obtain a brown liquid, which was purified by flash chromatography (pentane/Et₂O, 3:1) to afford **SA-2** (5.74 g, 37.70 mmol, 65%) as a pale-yellow oil.

**General data:** C_8_H_12_OSi; FW: 152.265; **TLC**: R_f_=0.5 (3:1 Pen/Et_2_O); UV (+); Vanillin: dark blue.

**^1^H-NMR** (600 MHz, CDCl_3_) δ 4.32 (s, 2H), 2.16 – 2.09 (m, 1H), 0.20 (s, 9H).

**^13^C-NMR** (151 MHz, CDCl_3_) δ 87.87, 87.24, 75.87, 70.74, 51.46, -0.39. (In agreement with lit. [27]).

**(E)-pent-2-en-4-yn-1-ol (SA-3)^[15][26]^**

To a solution of **SA-2** (5.50 g, 36.13 mmol, 1.0 equiv) in dry Et₂O (85 mL), LiAlH₄ (4.1 g, 108.31 mmol, 3.0 equiv) was added portion-wise at 0 °C. The resulting gray suspension was warmed to room temperature and stirred for 2 hours. The reaction was then cooled to 0 °C, and water (20 mL) was added dropwise, followed by 15% aqueous NaOH (10 mL). The suspension was allowed to warm to room temperature and stirred overnight. The mixture was filtered over a pad of Celite and washed with Et₂O. The filtrate was dried over MgSO₄, filtered, and concentrated under reduced pressure (rt, 400 mbar) to yield an orange solution. The crude residue was purified by flash chromatography (pure pentane to pentane/Et₂O, 1:1), affording **SA-3** (2.78 g, 22.40 mmol, 62%) as a yellow liquid. Due to the high volatility of SA-3, the solvent was not completely removed after flash chromatography, and the yield was determined by ¹H NMR.

**General data:** C_5_H_6_O; FW: 82.1; **TLC**: R_f_=0.4 (3:1 Pen/Et_2_O); UV (+); Vanillin: dark blue.

**^1^H-NMR** (600 MHz, CDCl_3_) δ 6.35 (dt, *J* = 16.0, 5.0 Hz, 1H), 5.74 (dq, *J* = 16.1, 2.1 Hz), 4.22 (ddd, *J* = 4.3, 1.4, 0.7 Hz, 2H), 2.89 (dd, *J* = 1.5, 0.7 Hz, 1H).

**^13^C-NMR** (151 MHz, CDCl_3_) δ 143.98, 109.17, 81.72, 77.96, 62.74.

**(2E,5E)-6-(tributylstannyl)hexa-2,5-dien-1-ol (19) ^[15][27]^**

To a solution of **CuCN (2.68 g, 29.90 mmol, 1.15 equiv)** in **dry THF (60 mL)**, **n-BuLi (2.5 M in hexane, 24 mL, 60.06 mmol, 2.30 equiv)** was added dropwise at **-78 °C**. The resulting suspension was allowed to warm slowly to **room temperature**. The reaction mixture was then cooled again to **-78 °C**, and **n-Bu₃SnH (16.2 mL, 60.06 mmol, 2.30 equiv)** was added dropwise. The resulting deep yellow solution was stirred at **-78 °C for 10 min**. Then, a solution of **SA-3 (2.60 g, 20.96 mmol, 1.0 equiv)** in **hexane (50 mL)** was added dropwise. The resulting deep orange solution was stirred at **-78 °C**. After 90 **min**, **aq. sat. NH₄Cl (540 mL)** and **25% aq. NH₄OH (60 mL)** were added, followed by **Et₂O (100 mL)**, and the layers were separated. The aqueous layer was extracted with **Et₂O (3×50 mL)**. The combined organic layers, dried over **MgSO₄**, filtered, and concentrated under reduced pressure to obtain **a yellow liquid**. The crude residue was purified by **flash chromatography (Hex/Et₂O/NEt₃, 90:10:1 to 50:50:1)** to afford **19 (2.31 g, 16.75 mmol, 80%)** as a pale-yellow oil.

**General data:** C_17_H_34_OSn; FW: 373.1617; **TLC**: R_f_=0.37 (3:1 Pen/Et_2_O); UV (+); Vanillin: dark blue.

**^1^H-NMR** (600 MHz, C_6_D_6_) δ 6.76 – 6.68 (m, 1H), 6.35 – 6.21 (m, 2H), 5.67 – 5.60 (m, 1H), 3.91 (s, 2H), 3.38 (dd, *J* = 6.2, 2.4 Hz, 1H), 1.62 – 1.54 (m, 6H), 1.41 – 1.34 (m, 6H), 0.99 – 0.89 (m, 15H).

**^13^C-NMR** (151 MHz, C_6_D_6_) δ 147.19, 133.93, 133.42, 132.38, 62.77, 29.60, 27.73, 13.98, 9.82. (in agreement with lit.[15]).

**(2E,4E)-5-(tributylstannyl)penta-2,4-dienoic acid (20) ^[15]^**

To a solution of **19 (2 g, 11.61 mmol, 1.0 equiv)** in **dry DCM (60 mL)** was added **NMO (2 g, 16.07 mmol, 3.0 equiv)**, followed by **TPAP (307 mg, 1.16 mmol, 8 mol%)** in a single addition. The resulting dark brown suspension was stirred at **room temperature for 3 h**. The reaction mixture was then filtered through a pad of **silica** and washed with **Et₂O**. The yellow filtrate was concentrated under reduced pressure to give **2.05 g of crude SA-4-1** as a brown oil.

**General data:** C_17_H_32_OSn; FW: 371.145; **TLC**: R_f_=0.53 (15:1 Pen/Et_2_O) UV (+); Vanillin: purple.

**^1^H NMR** (600 MHz, C_6_D_6_) δ 9.41 (d, *J* = 7.8 Hz, 1H), 6.78 – 6.57 (m, 2H), 6.44 (dd, *J* = 15.4, 9.9 Hz, 1H), 5.90 (dd, *J* = 15.3, 7.8 Hz, 1H), 1.61 – 1.48 (m, 6H), 1.44 – 1.25 (m, 6H), 1.02 – 0.87 (m, 15H).

**^13^C NMR** (151 MHz, C_6_D_6_) δ 192.90, 152.02, 148.78, 145.00, 29.45, 27.64, 13.91, 9.88.

**The crude aldehyde SA-4-1 was dissolved in t-BuOH (25 mL) and 2-methyl-2-butene (14 ml, 108.44 mmol, 20 equiv). The resulting light brown solution was cooled to 0 °C, and a suspension of NaClO₂ (4.00 g, 44.22 mmol, 8 equiv) in water (5.5 mL) and a suspension of NaH₂PO₄·2H₂O (3.45 g, 22.11 mmol, 4 equiv) in water (5.5 mL) were added. The resulting yellow solution was stirred at 0 °C for 1.5 h, then diluted with aq. sat. NH₄Cl (50 mL). Et₂O (50 mL) was added, and the layers were separated. The aqueous layer was extracted with Et₂O (2×50 mL). The organic layers were combined, dried over MgSO₄, filtered, and concentrated under reduced pressure to give 2.4 g of crude 20.**

**General data:** C_17_H_32_O_2_Sn; FW: 387.144; **TLC**: R_f_=0.47 (3:1 Pen/Et_2_O); UV (+); KMnO_4_.

**^1^H-NMR** (600 MHz, C_6_D_6_) δ 7.42 (dd, *J* = 15.3, 9.4 Hz, 1H), 6.71 – 6.53 (m, 2H), 5.77 (d, *J* = 15.2 Hz, 1H), 1.63 – 1.42 (m, 6H), 1.39 – 1.25 (m, 6H), 0.93 – 0.88 (m, 15H).

**^13^C-NMR** (151 MHz, C_6_D_6_) δ 179.81, 148.53, 148.34, 144.63, 119.80, 29.43, 27.64, 13.91, 9.81.

**
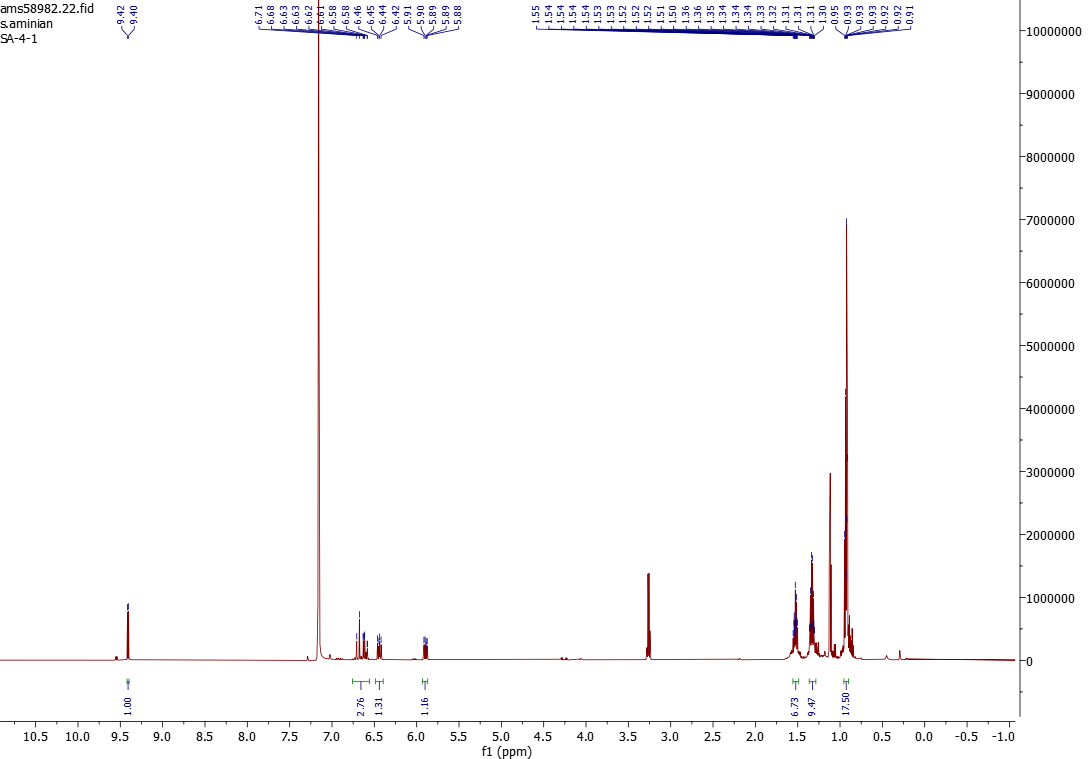

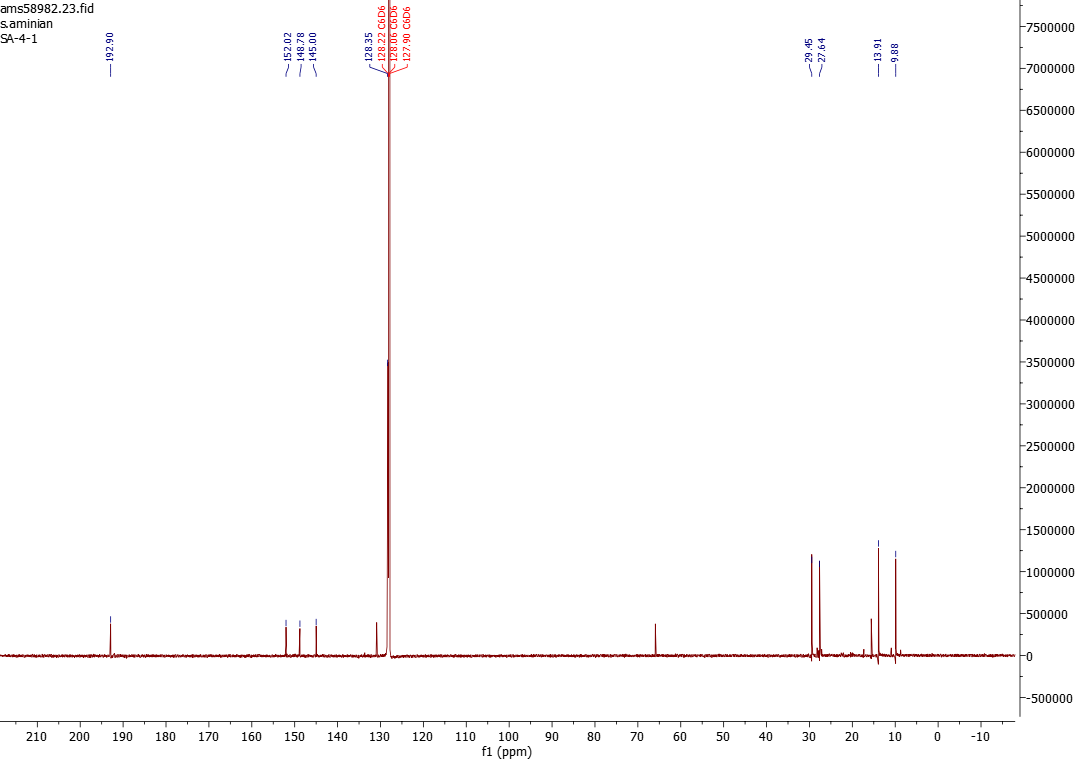
**

**(S)-methyl 3-hydroxy-2-((2E,4E)-5-(tributylstannyl)penta-2,4-dienamido)propanoate (22)**

To the solution of the crude carboxylic acid **20** (1 g, 2.58 mmol, 1.0 equiv) and L-serine methyl ester hydrochloride (475 mg, 3.10 mmol, 1.2 equiv) in dry THF (20 mL), was added DIPEA (1.1 mL, 6.19 mmol, 2.4 equiv). The resulting solution was cooled to 0 °C, and HBTU (1.18 g, 3.10 mmol, 1.2 equiv) was added to the solution in one portion. The resulting yellow suspension was stirred at 0 °C for 30 min and then allowed to warm to rt. After 4 h, the reaction mixture was diluted with aq. sat. NH_4_Cl (60 mL). Et₂O (60 mL) was added, and the layers were separated. The aqueous layer was extracted with Et₂O (3×50 mL). The combined organic layers were dried over MgSO₄, filtered, and concentrated under reduced pressure to give a crude residue. The crude product was purified by flash chromatography (Pentane/Et₂O/TEA, 1:1:0.1) to afford **22** (705 mg, 1.44mmol, 55% over 3 steps) as a pale-yellow oil.

**General data:** C_21_H_39_NO_4_Sn; FW: 488.248; TLC: R_f_=0.46 (Pen/Et_2_O 1:2); UV (+); KMnO_4_, ${[\alpha]}_{D}^{25}$= +3.778 (c = 0.45 in MeCN)

**^1^H-NMR** (400 MHz, C_6_D_6_) δ 7.55 – 7.41 (m, 1H), 6.81 – 6.47 (m, 3H), 5.44 (d, J = 14.9 Hz, 1H), 4.81 (dt, J = 7.3, 3.5 Hz, 1H), 3.91 – 3.77 (m, 2H), 3.30 (s, 3H), 3.00 (s, 1H), 1.67 – 1.50 (m, 6H), 1.44 – 1.30 (m, 6H), 1.01 – 0.89 (m, 15H).

**^13^C-NMR** (101 MHz, C_6_D_6_) δ 171.31, 166.31, 145.19, 144.54, 143.57, 123.04, 63.62, 55.45, 52.07, 29.508, 27.71 , 13.95 , 9.85.

**IR (ATR, cm⁻¹):** ṽ = 3343 (br), 2955 (m), 2924 (m), 2871 (m), 2851 (m) , 1742 (vs), 1649 (s), 1615 (m), 1562 (m), 1460 (m), 1438 (m), 1374 (m), 1209 (s), 1279 (w), 1209 (s), 1172 (s), 1072 (s), 1005 (s), 865 (m), 785 (w), 661(s), 595 (s), 510 (s) .

**HRMS** **(ESI^+^) *m/z*:** [M+Na]^+^ calcd for C_21_H_39_NO_4_SnNa: 512.1799, found: **512.1837.**

**methyl 2-((1E,3E)-4-(tributylstannyl)buta-1,3-dien-1-yl)oxazole-4-carboxylate (23)**

To a solution of **22** (600 mg, 1.23 mmol, 1.0 equiv) in dry DCM (10 mL) was added DAST (178 μL, 1.35 mmol, 1.1 equiv) dropwise at -78 °C. The resulting pale-yellow solution was stirred at -78 °C for 4 h. The reaction was quenched with K₂CO₃ (340 mg, 2.45 mmol, 2.0 equiv), allowed to warm to room temperature over 1 h, and then poured into sat. aq. NaHCO₃ (40 mL) and DCM (40 mL). The aqueous layer was extracted with DCM (3×30 mL). The combined organic layers, dried over MgSO₄, filtered, and concentrated under reduced pressure to give **594 mg** crude oxazoline as a pale yellow-brown oil.

**General data:** C_21_H_37_NO_3_Sn; FW: 470.23; TLC: R_f_=0.33 (Pen/Et_2_O 1:1); UV (+); KMnO_4_.

**^1^H NMR** (400 MHz, CD_3_CN) δ 6.97 – 6.89 (m, 1H), 6.84 – 6.65 (m, 2H), 6.02 (d, J = 15.5 Hz, 1H), 4.77 (dd, J = 10.1, 7.7 Hz, 1H), 4.46 – 4.40 (m, 2H), 3.71 (s, 3H), 1.63 – 1.43 (m, 6H), 1.38 – 1.24 (m, 6H), 1.01 – 0.85 (m, 15H).

**^13^C NMR** (101 MHz, CD_3_CN) δ 172.70, 166.42, 145.90, 145.63, 143.83, 117.34, 70.20, 69.47, 52.97, 29.79, 27.93, 13.96, 10.17.

**IR (ATR, cm⁻¹):** ṽ = 2955 (m), 2924 (m), 2871 (m), 2852 (m) (C–H), 1743 (vs) , 1645 (s), 1603 (m), 1561 (w), 1461 (m), 1438 (m), 1360 (s), 1287 (m), 1205 (s), 1176 (s), 1105 (w), 1071 (m), 1001 (vs), 956 (m), 916 (m), 863 (m), 770 (m), 662 (s) , 597 (m) , 508 (m), 486 (m), 452 (w).

**HRMS** (ESI^+^) *m/z*: [M+H] ^+^ calcd for C_21_H_38_NO_3_Sn: 472.1868, found: 472.1926**.**

The crude oxazoline (594 mg, 1.26 mmol, 1.0 equiv) was dissolved in dry DCM (10 mL). The resulting pale-yellow solution was cooled to 0 °C, and BrCCl₃ (504 μL, 5.05 mmol, 4.0 equiv) was added dropwise, followed by the addition of DBU (622 μL, 4.17 mmol, 3.3 equiv). The reaction mixture was stirred at 0 °C for 17 h, then diluted with hexane (10 mL) and directly purified by flash chromatography (Hex/EtOAc/NEt₃, 50:1:1 to 20:1:1) to afford **23** (507 mg, 1.08 mmol, 86% over 2 steps) as a pale-yellow oil.

**General data:** C_21_H_35_NO_3_Sn; FW: 468.217; TLC: R_f_=0.61 (Pen/Et_2_O 1:1); UV(+); KMNO_4_ .

**^1^H-NMR** (600 MHz, C_6_D_6_) δ 7.59 (s, 1H), 7.26-7.05 (m, 1H), 6.74 – 6.47 (m, 2H), 6.19 (d, J = 16 Hz, 1H), 3.45 (s, 3H), 1.60 – 1.53 (m, 6H), 1.38 – 1.31 (m, 6H), 0.98 – 0.91 (m, 15H).

**^13^C-NMR** (151 MHz, C_6_D_6_) δ 162.18, 161.55, 145.55, 143.49, 143.23, 140.20, 135.38, 115.72, 51.31, 29.50 , 27.68, 13.94 , 9.87.

**IR (ATR, cm⁻¹):** ṽ = 2955 (m), 2924 (m), 2851 (m), 2871 (w), 2851 (m), 1751 (s), 1727 (s), 1632 (m), 1575 (m), 1513 (m), 1469 (m), 1376 (w), 1318 (s), 1258 (m), 1194 (w), 1139 (m), 1113 (s), 1001 (vs), 945 (m), 862 (m), 803 (s), 760 (s), 690 (m), 661 (s), 596 (m), 509 (w).

**HRMS (ESI⁺)** ***m/z*: [M+H] ⁺ Calcd for** C_21_H_36_NO_3_Sn: 470.1712, found: 470.1759**.**

**2-((1E,3E)-4-(tributylstannyl)buta-1,3-dien-1-yl)oxazole-4-carboxylic acid (6)^[20]^**

To a solution of **23** (500 mg, 1.07 mmol, 1.0 equiv) in 1,2-dichloroethane (10 mL), trimethyltin hydroxide (770 mg, 4.24 mmol, 4.0 equiv) was added. The mixture was heated to 70 °C and stirred for 8 h. After completion, the reaction mixture was diluted with 0.2 M NaH₂PO₄·2H₂O (80 mL) and Et₂O (70 mL). The aqueous layer was extracted with Et₂O (2×50 mL). The organic layers were combined, dried over MgSO₄, filtered, and concentrated under reduced pressure to give 510 mg crude (contained residual solvent) **6** as a yellow wax, which was used as such in the couplings with alcohol **7**.

**General data:** C_20_H_33_NO_3_Sn; FW: 454.199; UV (+); KMNO_4_.

**^1^H-NMR** (400 MHz, CD_3_OD) δ 8.27 (s, 1H), 7.20 – 7.14 (m, 1H), 6.92 – 6.60 (m, 2H), 6.38 (d, J = 15.7 Hz, 1H), 1.60 – 1.52 (m, 6H), 1.39 – 1.33 (m, 6H), 0.94 – 0.88 (m, 15H).in agreement with lit.**[15]**

**^13^C NMR** (101 MHz, CD_3_OD) δ 163.43, 146.45, 144.66, 144.43, 141.51, 126.12, 115.86, 30.25, 28.31, 14.02, 10.40.

**HRMS (ESI⁺)** ***m/z*: [M+H] ⁺ Calcd for** C_20_H_34_NO_3_Sn: 456.1555 **found: 456.1554.**

**(1Z,4S,5R,6S,7E)-6-((tert-butyldimethylsilyl)oxy)-1-iodo-5-(methoxycarbonyl)-5-methylnona-1,7-dien-4-yl 2-((1E,3E)-4-(tributylstannyl)buta-1,3-dien-1-yl)oxazole-4-carboxylate (5)**

To the solution of alcohol **7** (27 mg, 0.0576 mmol, 1.0 equiv) and acid **6** (40 mg, 0.0864 mmol, 1.5 equiv) in dry CH_2_Cl_2_ (5 mL) under exclusion of light and inert atmosphere was add NEt_3_ (15 µl, 0.1056 mmol, 3.3 equiv), followed by DMAP (4.50 mg, 0.0346 mmol, 60 mol%) and 2-Methyl-6-nitrobenzoic anhydride (MNBA) (36.0 mg, 0.1037 mmol, 1.8 equiv) were added to the solution. The resulting yellow solution was stirred at rt for 18 h. aq. sat. NaHCO_3_ (10 mL) and CH_2_Cl_2_ (10 mL) were added, and the layers were separated. The aq. layer was extracted with CH_2_Cl_2_ (2x10 mL). The combined organic layers were dried over Na_2_SO_4_, filtered, and concentrated under reduced pressure. The crude residue was purified by flash chromatography (Hexane/EtOAc 10:1). The reaction yielded 25 mg (0.0276 mmol, 72% yield) of **5** as a pale-yellow oil. Additionally, 9 mg of the starting alcohol was recovered.

**General data:** C_38_H_64_NIO_6_SiSn; FW: 904.617; TLC: R_f_=0.62 (Hex/EA 8:1); UV (+); Vanillin: blue_,_ ${[\alpha]}_{D}^{25}$= +15.00 (c = 0.6 in MeOH)

**^1^H-NMR** (400 MHz, C_6_D_6_) δ 7.84 (s, 1H), 7.25 – 7.17 (m, 1H), 6.65 – 6.54 (m, 2H), 6.23 – 6.13 (m, 2H), 5.98 (dd, *J* = 9.7, 3.2 Hz, 1H), 5.88 (d, J = 7.3 Hz, 1H), 5.86 – 5.78 (m, 1H), 5.51 – 5.38 (m, 1H), 4.38 (d, *J* = 8.9 Hz, 1H), 3.37 (s, 3H), 2.82 – 2.70 (m, 2H) , 1.59 – 1.48 (m, 9H), 1.46 (s, 3H), 1.40 – 1.28 (m, 6H), 0.99 (s, 9H), 0.97 – 0.89 (m, 15H), 0.08 (s, 3H), 0.06 (s, 3H).

**^13^C-NMR** (101 MHz, C_6_D_6_) δ 173.01, 162.40, 160.16, 145.52, 143.90, 143.29, 140.27, 138.05, 135.26, 131.21, 129.74, 115.67, 84.90, 78.70, 74.81, 56.38, 51.46, 37.65, 29.50, 27.67, 26.18 , 18.36, 17.67, 15.60, 13.94, 9.87, -3.23, -4.72.

**IR (ATR, cm⁻¹):** ṽ = 2955 (m), 2926 (m), 2854 (m), 1743 (vs), 1632 (w), 1574 (m), 1461 (s), 1312 (m), 1250 (s), 1113 (vs), 1055 (s), 1000 (s), 836 (vs), 776 (s), 757 (m), 662 (m), 597 (m), 509 (w).

**HRMS (ESI⁺)** ***m/z***: [M+H] ⁺ Calcd for C_38_H_65_NIO_6_SiSn: 906.2643; found 906.2647.

**(2R,2'R,3S,3'S,4E,4'E)-dimethyl 2,2'-((4S,6Z,8E,10E,18S,20Z,22E,24E)-2,16-dioxo-3,13,17,27-tetraoxa-29,30-diazatricyclo[24.2.1.112,15]triaconta-1(28),6,8,10,12(30),14,20,22,24,26(29)-decaene-4,18-diyl)bis(3-((tert-butyldimethylsilyl)oxy)-2-methylhex-4-enoate) (24)**

A stock solution of Pd(PPh₃)₄ (10.8 mg, 9.24 μmol, 60 mol%), CuTC (17.5 mg, 92.4 μmol, 6.0 equiv), and [Ph₂PO₂][NBu₄]^26^ (44.6 mg, 97.02 μmol, 6.3 equiv) was prepared in dry DMF (2 mL) under an inert atmosphere. An aliquot (0.85 mL) of this red-brown solution was transferred to another flask, diluted with dry DMF (8 mL), thoroughly degassed, cooled to 0 °C, and kept in the dark. A degassed solution of **5** (15 mg, 15.4 μmol, 1.0 equiv) in dry DMF (2 mL) was added dropwise to the stirring reaction mixture at 0 °C. The reaction was stirred for 3 hours and 30 minutes at 0 °C, then filtered through a pad of silica and eluted with diethyl ether (2×80 mL). The two pale yellow filtrates were washed with water (80 mL), and the aqueous layer was extracted with diethyl ether (80 mL). The combined organic layers were washed again with water (2×80 mL), dried over anhydrous Na_2_SO₄, filtered and concentrated under reduced pressure to yield an orange oil. The crude residue was purified by flash chromatography (Hexane/EtOAc/TEA, 3:1:0.1) to afford the desired product **24** (6 mg, 6.15 μmol, 39%).

**General data:** C_54_H_74_N_2_O_12_Si_2_; FW: 975.3212; TLC: R_f_=0.58 (Hex/EA 3:1); UV (+); Vanillin: green_,_ ${[\alpha]}_{D}^{25}$= +115.111 (c = 0.45 in MeOH)

**^1^H-NMR** (600 MHz, CD_3_OD) δ 8.49 (s, 2H), 6.80 (br s, 2H), 6.69 (t, J = 13.2 Hz, 2H), 6.17 – 6.09 (m, 6H), 5.78 – 5.70 (m, 4H), 5.64 (q, J = 7.2 Hz, 2H), 5.44 (d, J = 10.1 Hz, 2H), 4.36 – 4.32 (dd, *J* = 10.3, 7.2 Hz, 2H), 3.59 (s, 6H), 2.70 (q, J = 11.1 Hz, 2H), 2.50 (q, J = 8.2 Hz, 2H), 1.78 (d, J = 4.9 Hz, 6H), 1.78 (s, 6H), 0.88 (s, 18H), 0.07 (s, 6H), 0.02 (s, 6H).

**^13^C NMR** (151 MHz, CD_3_OD) δ 174.83 , 163.44 , 160.57, 145.56, 139.04, 135.82, 135.49, 133.21, 132.15, 131.99, 131.39, 131.12, 116.12, 79.97, 76.79, 57.39, 52.22, 30.90, 26.31, 18.91, 17.99, 15.24, -3.25, -4.72.

**IR (ATR, cm⁻¹):** ṽ = 3055 (w), 2954 (m), 2928 (m), 2857 (m), 1734 (vs), 1614 (m), 1539 (w), 1462 (m), 1309 (w), 1247 (s), 1112 (s), 1055 (m), 996 (m), 836 (s), 777 (s), 751 (m).

**HRMS (ESI⁺)** ***m/z***: [M+NH_4_] ⁺ Calcd for C_54_H_78_N_3_O_12_Si_2_: 992.5118; found: 992.5124

**(2S,2'S,3S,3'S,4E,4'E)-dimethyl 2,2'-((4S,6Z,8E,10E,18S,20Z,22E,24E)-2,16-dioxo-3,13,17,27-tetraoxa-29,30-diazatricyclo[24.2.1.112,15]triaconta-1(28),6,8,10,12(30),14,20,22,24,26(29)-decaene-4,18-diyl)bis(3-hydroxy-2-methylhex-4-enoate)) (4)**

To a solution of **24** (6 mg, 6.15 μmol, 1.0 equiv) in dry THF (1200 μL) and dry pyridine (396 μL, 4.92 mmol, 800 equiv) at 0 °C in a 10 mL plastic centrifuge tube, HF∙py (70% HF, 383 μL, 14.76 mmol, 2400 equiv) was added dropwise under light exclusion. After 40 minutes from the completion of the addition, the reaction mixture was allowed to warm to room temperature and stirred for a total of 20 hours before stopping. The resulting pale-yellow solution was diluted with EtOAc (40 mL) and saturated aqueous NaHCO₃ (40 mL). The aqueous layer was extracted with EtOAc (2×40 mL), and the combined organic layers were washed with saturated aqueous NaHCO₃ (40 mL), dried over Na_2_SO₄, filtered, and concentrated under reduced pressure. The crude residue was purified by flash chromatography (Hexane/EtOAc, 5:1 to 1:1) to afford the desired product, disorazole Z1 **4** (2 mg, 2.67 μmol, 43%), along with recovered **24** (1 mg, 1.03 μmol, 16%) and mono-TBS-protected **24** (2 mg, 2.32 μmol, 37%).

**General data:** C_40_H_46_N_2_O_12_; FW: 746.7994; TLC: R_f_=0.25 (Hex/EA 1:1); UV (+); Vanillin: green_,_ ${[\alpha]}_{D}^{25}$=+58.000 (c = 0.1 in MeOH).

**^1^H-NMR** (600 MHz, CD_3_OD) δ 8.50 (s, 2H), 6.77 (br t, J= 18 Hz, 2H), 6.66 (t, J = 12 Hz, 2H), 6.15 – 6.11 (m, 6H), 5.77 – 5.72 (m, 2H), 5.72 – 5.67 (m, 2H), 5.64 (ddq, J = 15.3, 7.8, 1.5 Hz, 2H), 5.44 (d, J = 10.3 Hz, 2H), 4.33 (d, J = 7.7 Hz, 2H), 3.62 (s, 6H), 3.61-3.53 (m, 2H), 2.68 – 2.58 (m, 4H), 1.74 (dd, J = 6.4, 1.6 Hz, 6H), 1.39 (s, 6H).

**^13^C-NMR** (151 MHz, CD_3_OD) δ 175.03, 163.42, 160.58, 145.53, 139.01, 135.92, 135.53, 133.20, 132.05, 131.56, 131.28, 130.21, 116.09, 77.27, 76.53, 56.74, 52.30, 30.34, 18.02, 13.55.

**IR (ATR, cm⁻¹):** ṽ = 3470 (br), 2926 (m), 2855 (m), 1727 (vs), 1614 (m), 1518 (m), 1310 (s), 1227 (s), 1133 (s), 1113 (vs), 955 (vs), 970 (s), 756 (m), 716 (m).

**HRMS (ESI⁺)** ***m/z***: [M+NH_4_] ⁺ Calcd for C_40_H_50_N_3_O_12_: 764.3389; found: 764.3372**.**

Figure 1 Comparison of ¹H NMR and ¹³C NMR Spectra of Natural Disorazole Z1 (Top/green) ^[[1]](#footnote-1)^ and Synthesized Disorazole Z1 (Bottom/red) in methanol-d_4_ (600/150 MHz).

|  | **This work** | **Natural product** |  |
| --- | --- | --- | --- |
| NMR  field [MHz] | 150 | 150 |  |
| Solvent | CD_3_OD | CD_3_OD |  |
| **Carbon** | **δ [ppm]** | **δ [ppm]** | **Δδ** |
| 1/13 | 160.58 | 160.58 | - |
| 2/14 | 135.92 | 135.92 | - |
| 3/15 | 145.53 | 145.53 | - |
| 4/16 | 163.42 | 163.42 | - |
| 5/17 | 116.09 | 116.09 | - |
| 6/18 | 139.01 | 139.01 | - |
| 7/19 | 135.53 | 135.53 | - |
| 8/20 | 133.20 | 133.20 | - |
| 9/21 | 131.56 | 131.56 | - |
| 10/22 | 132.05 | 132.05 | - |
| 11/23 | 30.34 | 30.34 | - |
| 12/24 | 77.27 | 77.27 | - |
| 25/33 | 52.30 | 52.30 | - |
| 26/34 | 76.53 | 76.53 | - |
| 27/35 | 130.21 | 130.21 | - |
| 28/36 | 131.28 | 131.28 | - |
| 29/37 | 18.02 | 18.02 | - |
| 30/38 | 13.55 | 13.55 | - |
| 31/39 | 175.03 | 175.03 | - |
| 32/40 | 56.74 | 56.74 | - |

Table S1 ¹³C NMR Comparison of Natural Disorazole Z1 and Synthesized Disorazole Z1.

Table S2 ¹H NMR Comparison of Natural Disorazole Z1 and Synthesized Disorazole Z1.

|  | **This** **work** | | | **Natural Product** | | |  |
| --- | --- | --- | --- | --- | --- | --- | --- |
| NMR  field [M Hz] | 600 | | | 600 | | |  |
| Solvent | CD3OD | | | CD3OD | | |  |
| **Proton** | **δ [ppm]** | **Multiplicity** | ***J*** **[Hz]** | **δ [ppm]** | **Multiplicity** | ***J*** **[Hz]** | **Δδ** |
| 3/15 | 8.50 | s | - | 8.50 | s | - | - |
| 5/17 | 6.15 – 6.11 | m^a^ | - | 6.15 – 6.11 | m^a^ | - | - |
| 6/18 | 6.77 | br t | 18 | 6.77 | br t | 18 | - |
| 7/19 | 6.15 – 6.11 | m^a^ | - | 6.15 – 6.11 | m^a^ | - | - |
| 8/20 | 6.66 | t | 12 | 6.66 | t | 12 | - |
| 9/21 | 6.15 – 6.11 | m^a^ | - | 6.15 – 6.11 | m^a^ | - | - |
| 10/22 | 5.72 – 5.67 | m | - | 5.72 – 5.67 | m | - | - |
| 11/23 | 2.68 – 2.58 | m | - | 2.68 – 2.58 | m | - | - |
| 12/24 | 5.44 | d | 10.3 | 5.44 | d | 10.3 | - |
| 26/34 | 4.33 | d | 7.7 | 4.33 | d | 7.7 | - |
| 27/35 | 5.64 | ddq | 15.3, 7.8, 1.5 | 5.64 | ddq | 15.3, 7.8, 1.5 | - |
| 28/36 | 5.72 | m | - | 5.72 | m | - | - |
| 29/37 | 1.74 | dd | 6.4, 1.6 | 1.74 | dd | 6.4, 1.6 | - |
| 30/38 | 1.39 | s | - | 1.39 | s | - | - |
| 32/40 | 3.62 | s | - | 3.62 | s | - | - |
| 26/34-OH | 3.61-3.53 | m | - | 3.61-3.53 | m | - | - |

^a^ with , 5/17 , 7/19 , 9/21 .

References

[15] C. P. Bold; D. Lucena-Agell; M. Á. Oliva; J. F. Diaz; K.-H. Altmann *Angew. Chem. Int. Ed.* **2023***, 62, e202212190.*

[6] M. T. Crimmins; B. W. King; E. A. Tabet; K. Chaudhary *J. Org. Chem.* **2001**, *66*, 894-902.

[7] J. Wang, R. P. Hsung, S. K. Ghosh, *Org. Lett.* **2004**, *6,* 12, 1939-1942.

[24] S. P. Chavan, K. R. Harale, Tetrahedron Letters 2012, 53, 4683-4686.

[25] C. Herb, M. E. Maier, J. Org. Chem. 2003, 68, 8129-8135.

[26] S. J. Hein; D. Lehnherr; W. R. Dichtel *Chem. Sci.* **2017**, *8*, 5675–5681.

[27] C. Amatore; E. Blart; J. P. Genet; A. Jutand; S. Lemaire-Audoire; M Savignac *J. Org. Chem*. **1995**, *60*, 6829–6839.

[28] P. S. Ng; R. W. Bates; Tetrahedron **2016**, *72*, 6356–6362.

[29] H. Goossens; J. M. Winne; S. Wouters; L. Hermosilla; P. J. De Clercq; M. Waroquier; V. Van Speybroeck; S. Catak; *J. Org. Chem.* **2015**, *80*, 2609–2620.

[30] A. De Lera; V. Dominguez; US 6534545 B1, **2003**.

[31] N. Huwyler; K. Radkowski; S. M. Rummelt; A. Fürstner *Chem. Eur. J*. **2017**, *23*, 12412–12419.

1. The natural product sample was received directly from the Helmholtz Institute in Braunschweig and used as-is, without any further modification or purification. [↑](#footnote-ref-1)
